# Restricting Visual Exploration Directly Impedes Neural Activity, Functional Connectivity, and Memory

**DOI:** 10.1093/texcom/tgaa054

**Published:** 2020-08-25

**Authors:** Zhong-Xu Liu, R Shayna Rosenbaum, Jennifer D Ryan

**Affiliations:** Department of Behavioral Sciences, University of Michigan-Dearborn, Dearborn, Michigan 48128, USA; Rotman Research Institute, Baycrest Health Sciences, Toronto, ON M6A 2E1, Canada; Centre for Vision Research and Vision: Science to Applications (VISTA) Program, York University, Toronto, ON M3J 1P3, Canada; Rotman Research Institute, Baycrest Health Sciences, Toronto, ON M6A 2E1, Canada; Centre for Vision Research and Vision: Science to Applications (VISTA) Program, York University, Toronto, ON M3J 1P3, Canada; Department of Psychology, York University, Toronto, ON M3J 1P3, Canada; Rotman Research Institute, Baycrest Health Sciences, Toronto, ON M6A 2E1, Canada; Centre for Vision Research and Vision: Science to Applications (VISTA) Program, York University, Toronto, ON M3J 1P3, Canada; Departments of Psychology, Psychiatry, University of Toronto, Toronto, ON M5S 3G3, Canada

**Keywords:** functional connectivity/similarity, gaze fixations, hippocampus, neuroimaging, visual exploration

## Abstract

We move our eyes to explore the visual world, extract information, and create memories. The number of gaze fixations—the stops that the eyes make—has been shown to correlate with activity in the hippocampus, a region critical for memory, and with later recognition memory. Here, we combined eyetracking with fMRI to provide direct evidence for the relationships between gaze fixations, neural activity, and memory during scene viewing. Compared to free viewing, fixating a single location reduced: 1) subsequent memory, 2) neural activity along the ventral visual stream into the hippocampus, 3) neural similarity between effects of subsequent memory and visual exploration, and 4) functional connectivity among the hippocampus, parahippocampal place area, and other cortical regions. Gaze fixations were uniquely related to hippocampal activity, even after controlling for neural effects due to subsequent memory. Therefore, this study provides key causal evidence supporting the notion that the oculomotor and memory systems are intrinsically related at both the behavioral and neural level. Individual gaze fixations may provide the basic unit of information on which memory binding processes operate.

## Introduction

The oculomotor system may be a unique effector system that supports the development of memory (Ryan and Shen 2020). Key structures within the oculomotor and hippocampal (HPC) memory systems are phylogenetically old structures (Murray et al. 2017, 2018), and their shared evolutionary histories has resulted in a complex network of structural and functional connections between the 2 systems that span temporal, parietal, and frontal regions (Shen et al. 2016). By frequently moving the high-resolution fovea across the external world, rich visual details may be extracted, accumulated, and ultimately stored into a lasting memory representation (Yarbus 1967; Itti and Koch 2000; Henderson and Hayes 2017). Indeed, numerous eye tracking studies, across decades of research, have shown that gaze fixations—the discrete stops that are made by the eyes—predict subsequent memory, irrespective of the duration of viewing time (Loftus 1972; Chan et al. 2011; Damiano and Walther 2019). This relationship appears to causal, rather than merely correlational: restricting eye movements at encoding by having participants maintain central fixation results in a decrease in subsequent recognition memory performance (Henderson et al. 2005; Damiano and Walther 2019).

Findings from human neuroimaging provide further evidence of a relationship between the oculomotor and HPC memory systems. Neural activity in the HPC increases with an increasing number of gaze fixations (Liu et al. 2017), and this relationship is weaker in older adults who have smaller HPC volumes (Liu et al. 2018). Other work shows that HPC activity increases with decreasing fixation duration, presumably due to a higher rate of gaze fixations, although this was not assessed directly (Henderson and Choi 2015). However, the neuroimaging evidence to date is correlational in nature, as participants’ eye movements were measured under natural viewing conditions. If the relationship between fixations and HPC-mediated memory is direct, then modifying the rate and extent of visual exploration should modulate activity in the HPC as well as subsequent memory. Likewise, given the vast structural and functional network within which the oculomotor and HPC memory systems interact (Shen et al. 2016; Ryan, Shen, Kacollja, et al. 2020), changes in patterns of visual exploration should also affect either the set of regions that comprise functionally connected networks, or the strength of those functional connections.

In the present study, we experimentally manipulated participants’ viewing behavior and observed changes in the functional engagement of the HPC, surrounding medial temporal lobe, and ventral visual stream. Participants studied scenes and scrambled (color-tile) images either under *free-viewing* conditions, or they were asked to maintain fixation during viewing (*fixed*-*viewing*). Following scanning, participants were given a recognition memory task. Based on prior work, it was expected that the number of gaze fixations would relate to subsequent recognition memory, and that *fixed-viewing* would result in a decrease in recognition memory (Henderson et al. 2005; Damiano and Walther 2019). We also predicted that gaze fixations would be predictive of neural responses in the HPC, and in the parahippocampal place area (PPA) due to the use of scenes in the present task (Aguirre et al. 1996; Epstein and Kanwisher 1998; Epstein and Baker 2019). In comparison to *fixed-viewing*, *free-viewing* was predicted to result in increases in neural responses along the ventral visual stream and into the medial temporal lobe and HPC. Manipulating visual exploration was further predicted to change the configuration of functional networks and/or the degree to which regions were functionally connected during task performance. Finally, conjunction and cross-voxel similarity analyses were conducted to determine the extent to which the influence of gaze fixations on neural activity was similar to, or was unique from, activity indicative of successful encoding.

Alterations in the relationships between gaze fixations, neural activity in the HPC and broader medial temporal lobe, and subsequent memory due to viewing manipulations would suggest that visual exploration is a general mechanism that supports the binding of information into memory. Further evidence for the integral link between visual exploration and HPC function are expected to be found in patterns of brain activity that are similar across gaze modulation and subsequent memory effects. Unique effects of gaze fixations on HPC activity, above and beyond effects of subsequent memory, may suggest that gaze fixations provide the basic units of information on which the HPC operates.

## Materials and Methods

### Participants

Thirty-six healthy young adults (22 females; age: *M* = 23.58 years, *SD* = 4.17; education: *M* = 16.27 years, *SD* = 1.8) participated in exchange for monetary compensation. All participants had normal or corrected-to-normal vision (including color vision), and none had any neurological or psychological conditions. Participants were recruited from the University of Toronto and surrounding Toronto area community. All participants provided written informed consent. The study was approved by the Research Ethic Board at Rotman Research Institute at Baycrest Health Sciences.

### Stimuli

**Figure 1:**
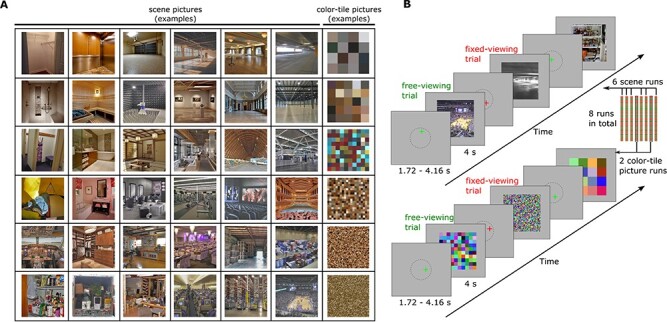
(*A*). Example stimuli. The scene images vary in clutter (along rows) and space size (along columns). The color-tile images vary in tile size. The clutter and size level were not relevant to the goals of the current study. (*B*). Scene processing task: Participants were presented with either a green or red fixation cross at the start of each encoding trial. A green fixation cross instructed the participants to freely view the upcoming scene or scrambled color-tile image (*free-viewing)*. A red fixation cross instructed the participants to maintain fixation on the location of the cross during the presentation of the upcoming scene or scrambled color-tile image (*fixed-viewing*). Participants were instructed to encode as much information regarding the scene or scrambled color-tile image as possible under both viewing conditions. 6 runs of scenes and 2 runs of scrambled color-tile images were presented to the participants, with timing as indicated.

Stimuli used in this experiment consisted of twenty-four color scene images (500 x 500 pixels; viewing angle: 7.95 x 7.95 degree) for each of 36 semantic scene categories (e.g., living room, arena, warehouse, etc.), for a total of 864 images. Half of the scene images were from a previous study by Park et al. (Park et al. 2015). The other half of the scene images were collected from Google Image using the same 36 semantic categories as the search terms. The 36 categories of scenes varied along 2 feature dimensions: the size of scene space and the clutter within the scene. Each feature dimension (size, clutter) had 6 levels in a balanced factorial design such that each level of one feature contained 6 levels of the other feature, resulting in 36 unique feature level combinations (for details, see (Park et al. 2015)). The 6 size levels “roughly follow a logarithmic scale based on the number of people the space may contain” and the 6 clutter levels were differentiated based on the quantity of individual elements included in the scenes, such as objects, people, etc. (for details, see Park et al. (2015), Experiment 2, page 1793). The newly collected images were selected to match the spatial size and clutter feature of the original images by (Park et al. 2015). Each newly collected picture was judged as being similar/consistent to the original pictures by at least 2 researchers. Example stimuli are presented in Figure 1*A*.

The 24 scene images in each scene category were randomly divided into 3 groups with 8 images in each group. One group of images (8 images/category x 36 categories = 288 images) was used for the *free-viewing* encoding condition, one group (288 images) for the *fixed-viewing* encoding condition, and one group (288 images) served as the lure images during the retrieval task. The assignment of the 3 groups of images to these experimental/stimulus conditions was counterbalanced across participants. Low-level features such as luminance and contrast were equalized across the 3 groups of images using a Matlab script based on the SHINE toolbox. Color histogram and spatial frequencies were calculated using Natural Image Statistical Toolbox for MATLAB (Bainbridge and Oliva 2015) and also balanced across the 3 groups of images.

The 8 images of each scene category in the free- and fixed-viewing conditions were randomly assigned to 8 fMRI encoding runs. Each run had 36 images (one scene from each category) that were viewed under *free-viewing* instructions and 36 images (one scene from each category) that were viewed under *fixed-viewing* instructions. Then, images from 2 randomly selected runs were scrambled, using 6 levels of tile sizes (i.e., 5, 10, 25, 50, 100, and 125 pixels) to resemble the cluster feature of the scene pictures. The pixels within each tile were averaged to create 2 runs of scrambled color-tile images (Figure 1*A*). Similarly, 2 images from each scene category that were to be used as lures in the retrieval phase were also scrambled to provide scrambled lure color-tile images for the retrieval task.

To summarize, 6 images x 36 categories = 216 scene images and 2 images x 36 categories = 72 color-tile images were used in the *free-viewing* encoding condition; the same number of images was used in the *fixed-viewing* encoding condition. All of the images from the encoding phase, together with an additional 6 images x 36 categories = 216 scene images and 2 images x 36 categories = 72 color-tile images, were used in the retrieval task.

The feature levels of the scene images (i.e., the size of scene space and the clutter within the scene) were fully balanced across the groups of images used during *free-viewing* and *fixed-viewing* encoding and retrieval images. For our current purposes, the focus was on the neural differences between the free viewing and fixed viewing encoding conditions; the feature level manipulation (size, clutter) had no effect on the current findings and was not considered in the current analyses.

### Procedure

As mentioned above, the encoding task had 8 runs; 6 runs contained images of scenes, and 2 runs contained color-tile images. Each run had 72 images; 36 of which were studied under free-viewing instructions, and 36 of which were studied under fixed-viewing instructions, with one image from each of the 36 scene categories for each viewing condition. The order of the 6 scene and 2 color-tile image runs was randomized for each participant.

During each encoding trial (see Figure 1), a fixation cross “+” was first presented for 1.72–4.16 seconds (exponential distribution with mean = 2.63 seconds), and participants were asked to fixate their eye gaze on the cross. The location of the cross was randomly determined within a radius of 100 pixels around the center of the scene image. The reason we varied the locations of the initial gaze fixations was to create variability in the to-be-foveated position across trials in the fixed-viewing condition. This was done to ensure that the effects of gaze fixation restriction were indeed due to the restriction of *visual exploration*, not the restriction of the eye gaze to a single *specific* location of visual field. Also, to avoid unwanted reduction of visual input in the fixed-viewing condition, which may occur if participants fixated on the edge of the images, initial fixations were set far from the edges of the images, that is, within a radius of 100 pixels around the center. The cross “+” could be presented in either a green or red color. After the fixation cross, a scene image was presented for 4 seconds. When the cross was green, participants were to freely explore the scene or color-tile image that followed in order to encode as much information as possible (i.e., the *free-viewing* condition). When the cross was red, participants were required to keep their eye gaze at the location of the fixation cross throughout the presentation of the image that followed (i.e., the *fixed-viewing* condition). The task for each run was 500 seconds long, with 10 seconds and 12.4 seconds added to the beginning and end of the task, respectively. The trials from the 2 viewing conditions were pseudo-randomized to obtain an adequate design efficiency by choosing the design with the best efficiency from 1000 randomizations using Matlab code by Spunt (Spunt 2019).

### Structural and Functional MRI

A 3 T Siemens MRI scanner with a standard 32-channel head coil was used to acquire structural and functional MRI images. T1-weighted high-resolution MRI images for structural scans were obtained using a standard 3D MPRAGE (magnetization-prepared rapid acquisition gradient echo) pulse sequence (170 slices; FOV = 256 x 256 mm; 192x256 matrix; 1 mm isotropic resolution, TE/TR = 2.22/200 ms, flip angle = 9 degrees, and scan time = 280 s). For the fMRI scan, the BOLD signal was assessed using T2^*^-weighted EPI acquisition protocol with TR = 2000 ms, TE = 27 ms, flip angle = 70 degrees, and FOV = 191 x 192 ms with 64 x 64 matrix (3 mm x 3 mm in-place resolution; slice thickness = 3.5 mm with no gap). Two hundred and fifty volumes were acquired for each fMRI run. Both structural and functional images were acquired in an oblique orientation 30° clockwise to the anterior–posterior commissure axis. Stimuli were presented with Experiment Builder (Eyelink 1000; SR Research) back projected to a screen (projector resolution: 1024x768) and viewed with a mirror mounted on the head coil.

### Eyetracking

Participants’ eye movements were recorded using an MRI-compatible eye tracker (Eyelink 1000; SR Research) with a sampling rate of 1000 Hz and spatial resolution of 1°. Calibration was done using the built-in EyeLink 9-point calibration procedure at the beginning of the experiment. Drift correction was performed between trials when necessary to ensure good tracking accuracy. Fixations and saccades were categorized using Eyelink’s default eye movement event parser. Specifically, a velocity threshold of 30°/s and acceleration threshold of 8000°/s were used to classify saccades (saccade onset threshold = 0.15°). Events not defined as saccades or blinks were classified as fixations. The number of fixations that participants made when they encoded each scene was calculated and exported using the EyeLink software Data Viewer.

### fMRI Data Preprocessing

SPM 12 (Statistical Parametric Mapping, Welcome Trust Center for Neuroimaging, University College London, UK; https://www.fil.ion.ucl.ac.uk/spm/software/spm12/ Version: 7487) in the Matlab environment (The MathWorks, Natick, USA) was used to process the functional images. Following the standard SPM 12 preprocessing procedure, slice timing was first corrected using sinc interpolation with the midpoint slice as the reference slice. Then, all functional images were aligned using a 6-parameter linear transformation. Next, for each participant, functional image movement parameters obtained from the alignment procedure, as well as the global signal intensity of these images, were checked manually using the freely available toolbox ART (http://www.nitrc.org/projects/artifact_detect/) to detect volumes with excessive movement and abrupt signal changes. Volumes indicated as outliers by ART default criteria were excluded later from statistical analyses. Anatomical images were co-registered to the aligned functional images and segmented into white matter (WM), gray matter (GM), cerebrospinal fluid (CSF), skull/bones, and soft tissues using SPM 12 default 6-tissue probability maps. These segmented images were then used to calculate the transformation parameters mapping from the individuals’ native space to the MNI template space. The resulting transformation parameters were used to transform all functional and structural images to the MNI template. For each participant, the quality of co-registration and normalization was checked manually and confirmed by 2 research assistants. The functional images were finally resampled at 2x2x2 mm resolution and smoothed using a Gaussian kernel with an FWHM of 6 mm. The first 5 fMRI volumes from each run were discarded to allow the magnetization to stabilize to a steady state, resulting in 245 volumes in each run.

### fMRI Analysis

#### Activation Differences: Free- versus Fixed-Viewing

We used SPM 12 to conduct the first (i.e., individual) level whole brain voxel-wise General Linear Model (GLM) analysis to examine brain activation differences between the *free-viewing* and *fixed-viewing* encoding conditions for both the scenes and color-tile pictures. In this event-related design, we separately convolved the onset of trials in the free-viewing and fixed-viewing condition with the canonical hemodynamic function (HRF) in SPM 12, which served to be the 2 main regressors of interest. Because there were runs of scenes, and runs of color-tile images, we had 4 regressors of interest: free-viewing of scenes, fixed-viewing of scenes, free-viewing of color-tile pictures, and fixed-viewing of color-tile pictures. Motion parameters (6 from SPM realignment, 1 from ART processing), as well as outlier volumes, were added as regressors of no interest. Default high-pass filter with cut-off of 128 s was applied. A first-order autoregressive model AR(1) was used to account for the serial correlation in fMRI time-series in the restricted maximum-likelihood estimation of the GLM. To examine the main question of whether brain activation was different for the *free-* and *fixed-viewing* condition, we constructed a *t* contrast to directly compare the 2 conditions ([*free-viewing—fixed-viewing*]) for each run and then averaged all 6 scene runs (note that one participant only had 5 scene runs). We did a similar analysis to compare the *free-* and *fixed-viewing* conditions for the color-tile pictures. We also constructed a *t* contrast to compare the viewing effect (i.e., the difference between the 2 viewing conditions) between the 2 types of stimuli (scenes versus color-tile pictures).

Because we had specific *a priori* brain regions of interest, that is, the HPC and parahippocampal place area (PPA), at the second (i.e., group) level we used a region of interest (ROI; For ROI definition, see ROI section) analysis approach. Specifically, we extracted, for each participant, the mean beta estimates within each ROI for each contrast (effect) and tested the ROI group effect using one-sample *t-*tests. Because the HPC has an elongated shape and it is likely that not all segments along the HPC longitudinal axis would show the same effect, we also examined the voxel-wised results within HPC, using all voxels in the HPC masks to perform the family-wise multiple comparison error correction (Threshold: *p*_fwe-corr_ = 0.05). SPM 12’s small-volume-correction procedure and the HPC MNI template masks were used in this analysis.

#### Parametric Modulation Analysis

In order to replicate our prior work (Liu et al. 2017, 2018) with face stimuli, we conducted a parametric modulation analysis in SPM 12 for the *free-viewing* scene condition to examine whether increases in the number of gaze fixations was associated with stronger activation in the brain’s memory and perceptual processing regions. Specifically, in addition to regressors in the design matrix described in the previous section, we added a linear parametric modulation regressor that consisted of the trial-wise number of fixations convolved with the canonical HRF. Although we added this regressor for all 4 conditions in the design matrix, the focus here is on the *free-viewing* scene condition based on our prior findings (Liu et al. 2017, 2018). The effect of the fixation modulation regressor in the *free-viewing* condition was averaged across all scene runs and carried to the second (i.e., group) level ROI analysis. The same approach for the second level analysis, as mentioned earlier, was used for this analysis.

To understand the extent to which there was similar modulation of brain activity by the number of fixations and by subsequent memory, we also used parametric modulation analysis to examine subsequent memory effects. We coded subsequent recognition memory for each encoding trial based on participants’ hit/miss response and confidence. Specifically, we assigned 2 points to stimuli that were correctly recognized as previously viewed with high confidence, 1 point for those correctly recognized with low confidence, 0 points for previously viewed images incorrectly endorsed as “new” with low confidence, and − 1 point for previously viewed images that were incorrectly endorsed as “new” with high confidence to construct the linear parametric modulator. The design matrix, contrasts, and the first and second level analysis procedures were identical to those used in the fixation modulation analysis, save for the use of different modulation regressors.

To test whether the modulation effect of the trial-wise number of fixations on HPC and PPA activity in the *free-viewing* scene condition was associated with the brain activity that supports subsequent memory, we did a conjunction analysis in which we thresholded the voxel-wise fixation modulation effects and subsequent memory effects at *p* = 0.05, with 50-voxel extension (no corrections) and examined the conjunction (i.e., overlap) map between the 2 modulation effects. This resulted a conjunction effect map with *p* < 0.0025, 50 voxel-extension (no correction).

In addition to focusing on the overlap between 2 modulation effects at the mean level, we also investigated cross-voxel similarity between the subsequent memory effect and the modulation effect of the trial-wise number of fixations in the HPC and PPA. This was done to test whether the brain activity associated with 2 behavioral variables (i.e., trial-wise number of fixation and subsequent memory) also share similar brain activity pattern. Specifically, for each participant, we extracted unthresholded first-level subsequent memory and fixation modulation effect (i.e., the first level GLM beta estimates) from each voxel in the HPC and PPA ROIs. These voxel-wise beta values from the 2 parametrical modulation analyses were vectorized for each ROI. Then, Pearson correlation was calculated between the 2 vectors and then Fisher’s Z transformed to reflect the cross-voxel pattern similarity between the subsequent memory effect and the modulation effect of the number of fixations. We obtained the similarity measure for both the scene *free-viewing* and *fixed-viewing* conditions and compared them at the group level. If the brain activity pattern modulated by the number of fixations are indeed important for supporting the subsequent memory, we should observe greater cross-voxel similarity in the *free-viewing* than the *fixed-viewing* condition. This would provide further evidence supporting that visual exploration and memory formation share similar brain mechanisms.

Finally, we examined whether the trial-wise number of fixations still predicted activity in the HPC after controlling for the effect of subsequent memory. We reasoned that if visual exploration is generally important for HPC processes, irrespective of subsequent conscious awareness, the trial-wise number of fixations should still predict the brain activity in the medial temporal lobe after the shared variance with subsequent memory was partialed out. To test this hypothesis, we simultaneously entered the 2 parametric modulation regressors, that is, trial-wise subsequent memory and number of fixations, to the same parametric modulation analysis (with SPM 12 regressor orthogonalization turned off). Then, we examined the modulation effects of the number of fixations without the contribution of the shared variance with subsequent memory. In this analysis, we used the identical first and second level analysis procedure as mentioned above (except for using 2 parametric modulators) and focused on the *free-viewing* condition for scenes.

One participant did not finish the retrieval task and was excluded from all parametric modulation analyses. 2 additional participants were excluded from the parametric modulation analyses where the color-tile picture condition was involved due to low eye movement data quality (> 10 trials with zero fixations).

#### PsychoPhysiological Interaction (PPI) Analysis

To investigate how brain connectivity may differ between the *free*- and *fixed-viewing* condition, we conducted a generalized psychophysiological interaction analysis (gPPI) (Friston et al. 1997; McLaren et al. 2012) using the PPAs as seed regions. We chose the PPAs as the seed regions because the PPA is closely connected with visual processing regions earlier in the ventral visual stream, as well as the HPC, which is critical for memory (Libby et al. 2012; Maass et al. 2015). Using the PPA as the seed region allowed us to examine how the connectivity among perceptual processing and memory regions in the brain can be modulated by different viewing conditions. We conducted the gPPI analysis using CONN toolbox v.18 (https://www.nitrc.org/projects/conn) in Matlab (Whitfield-Gabrieli and Nieto-Castanon 2012). First, all preprocessed functional images using SPM 12 were further preprocessed to eliminate signals that may affect the connectivity analysis. BOLD signal from the WM and CSF were used to regress out non-specific variance from the fMRI time series using a principal component-based noise correction method (Behzadi et al. 2007) as implemented in CONN toolbox. The first 5 principal components extracted from the WM and CSF were used in this noise removal procedure. Motion parameters obtained from the motion correction procedure were also used to regress out potential head movement effects. Slow fMIR signals were filtered out using 0.008 Hz high-pass filter. The data were also de-spiked using a hypobolic tangent function to reduce the impact of any potential outliers in the fMRI time series. After these further clean-up procedures, fMRI time series, averaged from the left and right PPA, were extracted from each encoding run. An interaction term was formed between the PPA time series and all other task condition regressors (which were convolved with the canonical HRF). Then, the original task condition regressors, PPA time series, and the interaction regressor were entered in the same GLM analysis. The contrast of the effect of the interaction regressor between the scene *free-viewing* and *fixed-viewing* conditions, reflecting the differences in PPA connectivity with other brain regions due to differences in viewing instructions, was the focus of this analysis. The results from all scene runs in each participant were averaged and carried to the second level group analysis. The same approach for the second level analysis, as mentioned earlier, was used for this analysis.

### ROI Definition

HPC ROIs were defined anatomically: The bilateral HPC masks in individual participants’ native space were first obtained using FreeSurfer *recon-all* function, version 6.0 (http://surfer.nmr.mgh.harvard.edu.myaccess.library.utoronto.ca) (Fischl 2012). Then, the same normalization parameters obtained from the SPM normalization procedure were used to transform these HPC masks into MNI normalized space. PPA ROIs were defined functionally: we first contrasted the scene and color-tile pictures conditions, collapsed over the *free-* and *fixed-viewing* conditions, at the individual-level analyses. Then, at the group level, we localized the maximumly activated cluster in the bilateral parahippocampal cortex at the threshold of *p*_fwe-corr_ = 0.05 (family-wise error multiple comparison correction). The MNI coordinates for the peak activation of the contrast were [32, −34, −18] for the right PPA and [−24, −46, −12] for the left PPA. The left and right PPA mask contained 293 and 454 voxels respectively (see Supplementary Figure S1).

### Statistical Thresholding

The threshold for statistical significance was set at *p* = 0.05 for the ROI analyses when the mean value of the estimated effect was obtained. For the whole HPC, when the results were not significant, *p*_fwe-corr_ = 0.05 (family-wise error multiple comparison correction) was used within the HPC (i.e., small volume correction) to examine whether voxel clusters in the HPC showed significant effects. We also present the whole-brain voxel-wise results thresholded at *p* = 0.005 with 10 voxel extension (uncorrected) to facilitate future meta-analysis (Lieberman and Cunningham 2009). The automated anatomical labeling (AAL) toolbox (Tzourio-Mazoyer et al. 2002) was used to identify the anatomical labels for regions that showed significant effects, which are reported in Supplementary Tables. Because our analyses were all *a priori* hypothesis driven, and to follow SPM12 analysis scheme, all *t* tests were one tailed.

### Post-fMRI Retrieval Task

After the fMRI encoding task, participants were given a 60-minute break before they began a retrieval task in a separate testing room. All scenes and color-tile images from the fMRI encoding task (36 images/condition/run x 2 conditions x 8 runs = 576 images) were tested in the retrieval task. In addition, 288 images that were not used in the encoding task (6 images x 36 categories =216 scene images and 2 images x 36 categories = 72 color-tile images) were also included as lures. The 864 images were divided into 6 blocks.

For each retrieval trial, a fixation cross “+” was presented for 1.5 seconds. For the previously viewed images (i.e., images from the encoding phase), the cross “+” was presented at the same location as it was during the encoding task. Following the presentation of the fixation cross, the retrieval image was presented on the screen for 4 seconds. Participants were given 3 seconds to indicate whether the image was a “new” or “old” (i.e., previously viewed during the fMRI task) image using 4 keys on the keyboard: z—high confidence “old”, x—low confidence “old”, n—high confidence “new”, and m—low confidence “new.” The response instruction was shown on the screen during the time window in which participants made their response. During the retrieval task, participants’ eye movements were recorded using the Eye-link II head mounted infrared camera system (SR Research Ltd, Oakville, ON, Canada) at a sampling rate of 500 Hz. However, the eye movement data from the retrieval task were not relevant for our current focus and are not discussed further here.

To measure recognition memory, we took participants’ response confidence into consideration and assigned 2 points to stimuli that were correctly recognized as previously viewed with high confidence, 1 point for those correctly recognized with low confidence, 0 points for previously viewed images incorrectly endorsed as “new” with low confidence, and − 1 point for previously viewed images that were incorrectly endorsed as “new” with high confidence. This recognition memory measure was then used to investigate the behavioral consequence of the manipulation of viewing condition, the relationship between the trial-wise number of fixations and memory performance, and brain regions supporting memory through subsequent memory analyses. For the participant who did not finish the retrieval task, available trials were used to calculate the memory performance.

## Results

### Eye Movements

**Figure 2:**
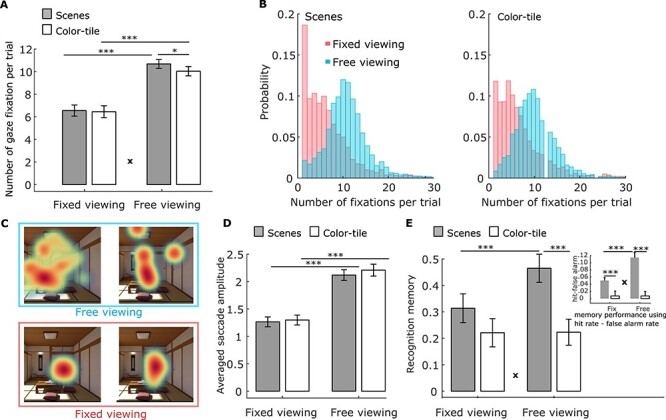
(*A*). Number of gaze fixations. Participants made more fixations on average per trial under *free-viewing* versus *fixed-viewing* instructions. More fixations were elicited by scenes versus color-tile pictures, but only in the *free-viewing* condition. (*B*). The distribution (across all participants) of the number of gaze fixations for the scenes (left) and color-tile pictures (right) under fixed (red) and free (blue) viewing conditions. Similar distributions are observed for the number of fixations for the scenes and color-tile pictures. (*C*). Fixation heatmaps (weighted by fixation durations) for a sample scene in the *free-viewing* versus *fixed-viewing* condition illustrates the difference in viewing patterns in the 2 conditions. (*D*). Average saccade amplitude, which is larger under *free-viewing* versus *fixed-viewing* instructions. (*E*). Recognition memory performance (with confidence considered) was better in the *free-viewing* versus *fixed-viewing* condition only for scenes. Recognition memory was calculated by assigning 2 points to stimuli that were correctly recognized with high confidence, 1 point for those correctly recognized with low confidence, 0 points for previously viewed images endorsed as “new” with low confidence, and − 1 point for previously viewed images endorsed as “new” with high confidence. The embedded bar graph shows memory accuracy results calculated using hit rate-false alarm rate. ^*^ = *p* < 0.05; ^***^ = *p* < 0.001. **x** = significant ANOVA interaction effect, *p* < 0.005, *t* test with *df* = 35. Error bars represent Standard Error.

To ensure that participants had complied with the viewing instructions, we compared the number of fixations (Figure 2*A*–*C*) and the average saccade amplitude (Figure 2*D*) in the *free-* and *fixed-viewing* conditions for the scenes and color-tile pictures. A 2x2 repeated measures ANOVA (viewing condition by stimulus type) revealed that participants made a greater number of gaze fixations (*F*(1,35) = 97.24, *p* < 0.0001), and had a larger saccade amplitude (*F*(1,35) = 80.53, *p* < 0.0001), in the *free-viewing* condition than the *fixed-viewing* condition, for both the scenes and the color-tile pictures. A significant interaction for the number of fixations only (*F*(1,35) = 8.78, *p* = 0.005) indicated that more gaze fixations were made to scenes versus color-tile pictures in the *free-viewing*, but not in the *fixed-viewing* condition.

### Memory Performance

A 2 x 2 repeated measures ANOVA on recognition memory performance (with confidence considered) revealed a significant interaction (*F*(1,35) = 27.85, *p* < 0.0001), indicating that recognition memory was significantly better in the *free-* versus *fixed-viewing* condition only for the scenes (Figure 2*E*). We also found a significant main effect of viewing condition (*F*(1,35) = 27.23, *p* < 0.0001) and stimulus type (*F*(1,35) = 6.92, *p* < 0.013; Figure 2*E*). Similar results were obtained when memory was measured using hit rate—false alarm rate such that the corrected accuracy was higher for scenes than for color-tile pictures (*F*(1,35) = 27.91, *p* < 0.0001), and higher for the images studied under *free-viewing* versus fixed-viewing instructions (*F*(1,35) = 21.35, *p* < 0.0001). This latter difference was significant only for the scenes (Interaction effect: *F*(1,35) = 22.91, *p* < 0.0001; See the embedded bar graph in Figure 2*E*). For detailed statistics, see Supplementary Table S1.

We then ran a linear regression analysis for each condition in each run using the trial-wise number of fixations to predict recognition memory (with confidence considered). The number of gaze fixations positively predicted recognition memory for the scenes (mean regression coefficient *β* = 0.011, *t(35)* = 2.75, *p* = 0.009 and *β* = 0.032, *t(35)* = 6.10, *p* < 0.0001 for the *fixed-* and *free-viewing* condition, respectively), but not for the color-tile pictures (*β* = −.0028 and − 0.0025, *t(35)* = −0.65 and − 0.48, *p* > 0.5 for the *fixed-* and *free-viewing* condition, respectively). The prediction was stronger for scenes studied under *free-* versus *fixed-viewing* instructions (*t(35)* = 3.51, *p* = 0.0013. See Supplementary Table S1).

### Neuroimaging Results

#### Brain Activation Differences between the Free- versus Fixed-Viewing Conditions

**Figure 3:**
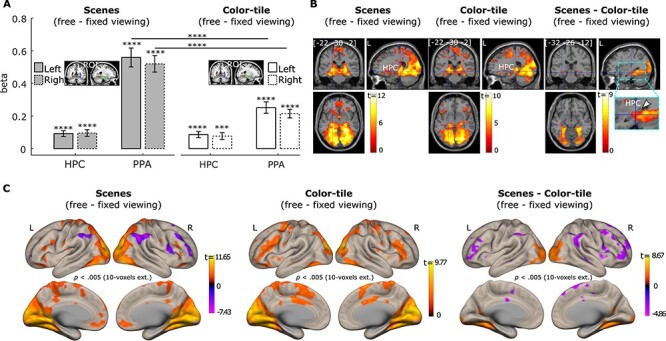
(*A*). Neural activity difference between the *free-* and *fixed-*viewing in the hippocampus (HPC) and parahippocampal place area (PPA) ROIs. Significant responses were observed in the HPC and PPA bilaterally for the *free*- versus *fixed*-viewing contrast. This *free-* versus *fixed* viewing difference was larger for scenes than for color-tile pictures in the PPA, but not in the HPC at the ROI level. *** = *p* < 0.001; **** = *p* < 0.0001. *t* test with *df* = 35, one-tailed. Error bars represent Standard Error. (*B*). Brain section views of the *free-* versus *fixed-* viewing activation differences for scenes (left), and color-tile pictures (middle). The scene (free-fixed) versus color-tile pictures (free-fixed) contrast, that is, the interaction effect, is depicted on the right. The zoomed-in image thresholded at *p* < 0.05 with no corrections shows the voxels within the HPC. The voxel at the crosshair survived Small Volume Correction within the HPC (*p*_fwe-corr_ < 0.05). (*C*). Surface views of the *free-* versus *fixed-* viewing activation differences for scenes (left), and color-tile pictures (middle). The scene (free-fixed) versus color-tile pictures (free-fixed) contrast is depicted on the right. The occipital cortex and ventral and medial temporal cortex showed stronger activation during the *free-viewing* condition, compared to the *fixed-viewing* condition. Medial temporal lobe regions and the PPA showed a stronger effect of viewing condition for the scenes compared to the color-tile pictures. For illustration purposes, data in B and C are thresholded at *p* = 0.005, 10-voxel extension with no corrections. For the brain section and surface views, L indicates the left hemisphere and R the right hemisphere.

To assess whether activity in the PPA and HPC was modulated by viewing instructions, we examined the brain activation contrast between the *free-* and *fixed-viewing* conditions for the scenes and color-tile pictures. The PPA and HPC showed stronger activation in the *free-viewing* condition than in the *fixed-viewing* condition for scenes (Figure 3*A*; *t(35)* = 5.20/4.67 and 9.63/10.32, for the left/right HPC and left/right PPA respectively, all *p* < 0.0001). Similar effects were found for the viewing of color-tile pictures (Figure 3*A*; *t(35)* = 4.61/3.64 and 7.16/8.17, for the left/right HPC and left/right PPA respectively, all *p* < 0.001). Directly comparing the effect of viewing condition for the scenes and color-tile pictures revealed that the *free*- versus *fixed-viewing* difference in activation for the left and right PPA was larger for scenes than color-tile pictures (*t(35)* = 6.25 and 6.79, *p* < 0.0001). When the whole HPC was used as the ROI, the effect of viewing condition did not differ between scenes and color-tile pictures (*t(35)* = 0.14 and 0.17, *p* = 0.80 and 0.51, for the left and right HPC, respectively).

However, we also examined whether any voxels inside the HPC showed differences in the effect of viewing condition for scenes versus color-tile pictures. Based on the whole brain voxel-wise results (*p* < 0.005 with 10 voxel extension) and the small-volume-correction analysis in SPM 12, we found that voxels in both the left and right MNI HPC masks showed a stronger viewing condition effect (*free-viewing* > *fixed-viewing*) for scenes compared to the color-tile pictures (*p*_fwe-corr_ = 0.022 with family-wise error correction, *p* < 0.0001 without correction, peak voxel location: [−34, −32, −12] for the left HPC, and *p*_fwe-corr_ = 0.024 with family-wise error correction, *p* < 0.0001 without correction, peak voxel location: [34, −34, −10], for the left HPC). Brain activation section and surface images (with *p* = 0.005, 10 voxel extension; no corrections) in Figure 3*B* and *C*, respectively, illustrate the effects in these ROIs and other brain regions. The whole brain voxel-wise results are also presented in Supplementary Table S2, S3, and S4.

#### Modulation of the Number of Gaze Fixations on Neural Responses

**Figure 4:**
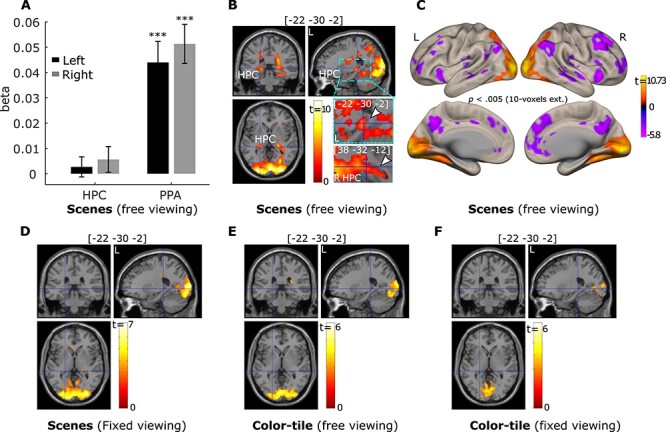
The number of gaze fixations positively predicts activation in the hippocampus (HPC) and parahippocampal place area (PPA) bilaterally. (*A*). Results for the anatomical HPC ROIs and functional PPA ROIs (*** = *p* < 0.001, *t* test with *df* = 34, one-tailed. Error bars represent Standard Error.). (*B*). Activation of a cluster in the bilateral HPC was positively predicted by the number of gaze fixations (*p* < 0.05 Small volume family-wise error correction). The left HPC: *p*_fwe-corr_ = 0.001, *p* < 0.00001, voxel size = 177 voxels with the peak voxel location = [−22, −30, −2]; for the right HPC: *p*_fwe-corr_ = 0.009, *p* < 0.00001, voxel size = 87 voxels with the peak voxel location = [28, −34, 4]. The zoomed-in image thresholded at *p* < 0.05 with no corrections shows the voxels within the HPC. The voxel at the crosshair survived Small Volume Correction within the HPC (*p*_fwe-corr_ < 0.05). (*C*). Brain surface views for the prediction of gaze fixations. (*D, E*), and (*F*) show the fixation modulation effects for the scenes under *fixed viewing*, color-tile pictures under *free-viewing*, and color-tile pictures under *fixed-viewing*, respectively. For illustration purposes, brain section (*B, D, E, F*) and surface (*C*) views are also presented at *p* < 0.005, 10-voxel extension with no corrections. For the brain section and surface views, L indicates the left hemisphere and R the right hemisphere.

To replicate our prior work (Liu et al. 2017, 2018), we investigated whether the trial-wise number of fixations was associated with HPC and PPA activation during *free-viewing* of scenes. Our parametric modulation ROI analysis showed that greater numbers of gaze fixations predicted stronger activation in the left and right PPA (*t(34)* = 5.31 and 6.64, *p* < 0.001). The effect was not significant for the HPC when the whole HPC anatomical ROIs were used (*t(34)* = 0.68 and 1.12, *p* = 0.50 and 0.27 for the left and right HPC respectively; Figure 4*A*). However, not all HPC regions along its longitudinal axis were affected by the viewing manipulation (Figure 3*B*). Therefore, based on the whole brain voxel-wise results (*p* < 0.005 with 10 voxel extension), we did a similar small volume correction analysis using the HPC MNI template masks to correct for multiple comparisons and found a cluster of voxels in the left and right HPC with peak voxels that survived family-wise error correction at *p*_fwe-corr_ = 0.05 (For the left HPC: *p*_fwe-corr_ = 0.001, *p* < 0.00001, voxel size = 177 voxels with the peak voxel location = [−22, −30, −2]; for the right HPC: *p*_fwe-corr_ = 0.009, *p* < 0.00001, voxel size = 87 voxels with the peak voxel location = [28, −34, 4]. A zoomed image is also presented in Figure 4*B* to illustrate the effects.). The whole brain voxel-wise results are presented in Figure 4C and Supplementary Table S5. Fixation modulation effects were not significant in HPC and PPA ROIs in the scene *fixed-viewing*, color-tile *free-viewing*, and color-tile *fixed-viewing* conditions (Figure 4*D*–*F*). Moreover, the prediction of the numbers of gaze fixations in the *free-viewing* condition was stronger for scenes than the color-tile pictures for both the left and right PPA (*t(32)* = 6.80 and 5.58, *p* < 0.001) and the left and right HPC (*t(32)* = 2.83 and 3.22, *p* = 0.004 and 0.0015). When visual exploration was restricted, the prediction of fixations on the HPC and PPA activation was also reduced to a larger extent for scenes than the color-tile pictures (*t(32)* = 2.81 and 3.22, *p* = 0.004 and 0.0015 for the left and right PPA, *t(32)* = 1.49 and 1.95, *p* = 0.07 and 0.03 for the left and right HPC).

#### P‌PA Connectivity Differences Due to Viewing Condition

**Figure 5:**
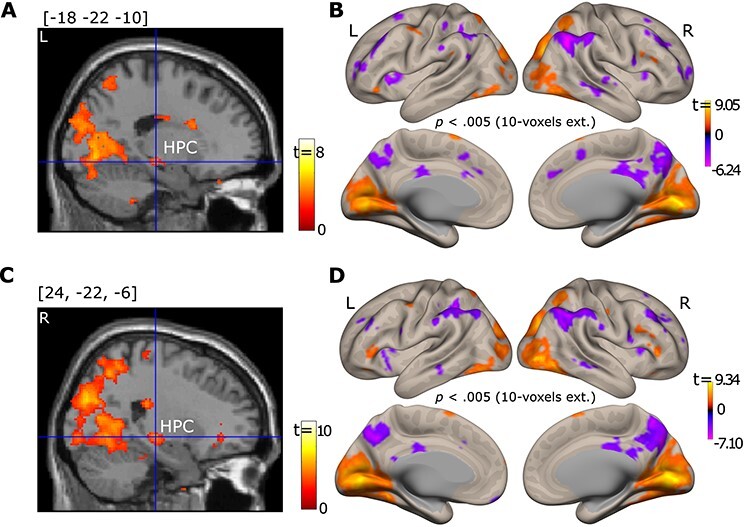
Brain section views of the left (*A*) and right (*C*) HPC, for which connectivity with the left (top) and right (bottom) PPA was modulated by viewing condition. Brain surface viewings of free versus fixed viewing modulation effect on functional connectivity for the left (*B*) and right (*D*) PPA. For illustration purposes, data were thresholded at *p* = 0.005, 10-voxel extension with no corrections. The left PPA showed connectivity with a cluster in the left HPC (voxel size = 20 voxels with the peak voxel location = [−22, −22, −10], *p*_fwe-corr_ = 0.016, *p* < 0.001), and the right PPA showed connectivity with a cluster in both the left HPC (voxel size = 58 voxels with the peak voxel location = [−22, −24, −8], *p*_fwe-corr_ = 0.001, *p* < 0.0001) and the right HPC (voxel size = 24 voxels with the peak voxel location = [26, −24, −8], *p*_fwe-corr_ = 0.014, *p* < 0.001) that was stronger in the *free-* than in the *fixed-viewing* condition. For the brain section and surface views, L indicates the left hemisphere and R the right hemisphere.

Given its role in the perceptual processing of scenes, we used the bilateral PPA as seeds to explore the effect of viewing condition on functional connectivity. Using the generalized psychophysiological interaction (gPPI) analysis implemented in the CONN toolbox, we found voxel clusters in the bilateral HPCs whose connectivity with the PPA was stronger in the *free-viewing* than *fixed-viewing* condition (Threshold: Small volume correction, *p*_fwe-corr_ < 0.05. Figure 5). Other regions, such as the lateral and medial occipital cortex and inferior and superior parietal lobules, also showed stronger connectivity with the PPAs in the *free-* versus *fixed-viewing* condition (The whole brain voxel-wise PPA connectivity results are also presented in Supplementary Table S6). We note that the voxel clusters in the HPC that showed stronger connectivity with PPA in the *free-viewing* than *fixed-viewing* condition were part of a larger voxel cluster in the lateral geniculate nucleus that showed this effect. Therefore, we cannot exclude the possibility that the effect in HPC may contain a spill-over effect from the lateral geniculate. In a follow-up analysis, we used individual participants’ HPC ROIs as seeds and repeated the connectivity analysis. Because the number of HPC mask voxels are much larger than the number of voxels in the LGN that can potentially contaminate the HPC, the potential influence of the LGN should be quite limited in this analysis. The results showed that the right HPC had a stronger connectivity with the right PPA in the *free-* then *fixed-viewing* condition (*t(35)* = 1.74, *p* = 0.045; For the effect of right HPC and left PPA connectivity, *t(35)* = 1.32, *p* = 0.098).

#### Modulation of the Number of Gaze Fixations and Subsequent Memory

**Figure 6:**
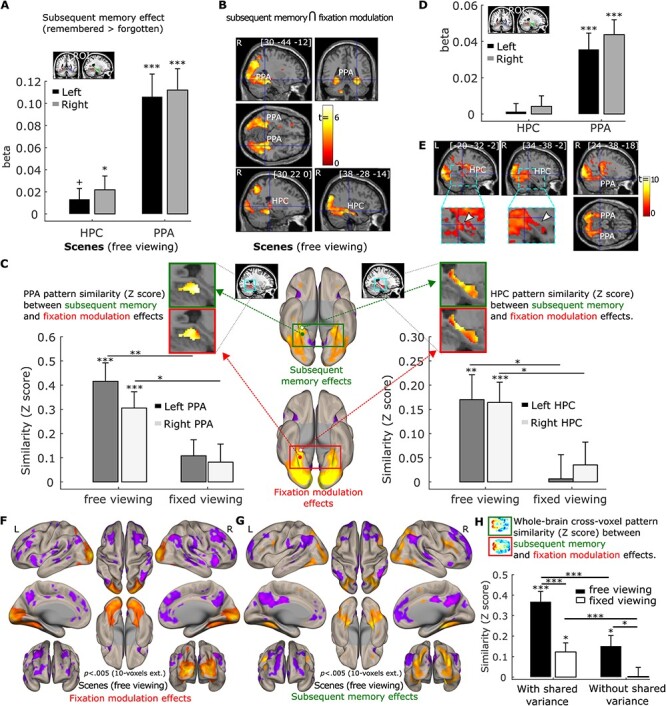
(*A*). Subsequent memory positively predicts activation in the hippocampus (HPC) and parahippocampal place area (PPA) bilaterally. ROI analysis results were based on anatomical HPC ROIs and functional PPA ROIs. (*B*). Activation in a cluster in the PPA bilaterally and in the right HPC was positively predicted by the number of gaze fixations and subsequent memory in a conjunction analysis (*p* = 0.0025, with 50-voxel extension, no corrections). (*C*). Cross-voxel pattern similarity between the fixation modulation effect and subsequent memory effect in HPC and PPA ROIs are greater in the scene *free-viewing* than the *fixed-viewing* condition. Ventral brain surface views of voxel-wise fixation modulation effects (left) and subsequent memory effect (right) showing the ventral and posterior medial temporal lobe regions are each associated with both the number of gaze fixations and subsequent memory performance. Voxel-wise fixation modulation and subsequent memory effect in HPC and PPA at the group level are embedded to illustrate the similarity between the 2 effects in the *free-viewing* condition. (*D*). The number of fixations predicted activation in PPA bilaterally after controlling for subsequent memory effect. (*E*). Activation of a cluster in the PPA and HPC bilaterally was positively predicted by the number of gaze fixations after controlling for subsequent memory effect (*p* = 0.005, with 10-voxel extension, no corrections). The zoomed-in images thresholded at *p* < 0.05 with no corrections show the voxels within the HPC. The voxel at the crosshair survived Small Volume Correction within the HPC (*p*_fwe-corr_ < 0.05). (*F*. & *G*). Brain surface views for the prediction of the number of gaze fixations (*F*) and subsequent memory (*G*) for the scenes under *free viewing* after controlling for shared variability between the 2 predictors. (*H*). The whole brain cross-voxel pattern similarity between the fixation modulation and subsequent memory is higher in the *free-* than *fixed-viewing* conditions for scenes, with or without controlling for the shared variability between the 2 variables. A horizontal section of the whole brain voxel-wise fixation modulation and subsequent memory effect at the group level are embedded to illustrate the similarity between the 2 effects in the *free-viewing* condition. Note: Surface and section view (*B, E, F*), and (*G*) are presented at *p* < 0.005, 10-voxel extension with no corrections. For the brain section and surface views, L indicates the left hemisphere and R the right hemisphere. For (*A, C, D*), and (*H*), + = *p* < 0.10; * = *p* < 0.05; ** = *p* < 0.005; *** = *p* < 0.001, *t* test with *df* = 34, one-tailed. Error bars represent Standard Error.

The relationship between the modulation effects of the number of gaze fixations and of subsequent memory performance in the *free-viewing* scene condition was examined. First, using parametrical modulation ROI analysis, we found that PPA activation was stronger for subsequently remembered than forgotten trials (*t(34)* = 5.17 and 5.81, *p* < 0.001; Figure 6*A*). The right HPC showed similar significant effects (*t(34)* = 1.75, *p* = 0.045; left HC, *t(34) =* 1.36, *p* = 0.092; Figure 6*A*). The whole brain voxel-wise subsequent memory effects are presented in Supplementary Table S7.

To confirm that the PPA and HPC were modulated by both trial-wise number of fixations and subsequent memory, we did a conjunction analysis in which we thresholded the gaze fixation and subsequent memory modulation effects at *p* = 0.05, with 50-voxel extension (no corrections) and examined the overlap. This revealed a conjunction effect with *p* < 0.0025, 50 voxel-extension (no correction) in which the right and left PPA and clusters of voxels in the right HPC (192 voxels) showed stronger activation when scenes were viewed with more fixations *and* were subsequently remembered (Figure 6*B*).

Although we found that the 2 behavioral variables (the number of fixations and subsequent memory) had shared neural variance, the conjunction analysis cannot test the extent to which there is a similar pattern of brain activity associated with the 2 behavioral variables. To confirm that the effect of the trial-wise number of fixations and subsequent memory had similar brain activation pattern, we conducted a pattern similarity analysis in which the cross-voxel (within the HPC and PPA) subsequent memory effect pattern of neural activity was correlated with the cross-voxel gaze fixation modulation effect pattern of neural activity. If visual exploration (as indexed by the number of fixations) is indeed associated with memory formation at the brain level in the *free-viewing* condition for scenes, the cross-voxel pattern of the trial-wise number of fixations modulation effects should be correlated with the cross-voxel brain activity pattern that reflects subsequent memory performance. As shown in Figure 6*C*, consistent with our hypothesis, the pattern similarity between the gaze fixation and subsequent memory modulation effects was significantly larger than zero for the left and right HPC (*t(34)* = 3.30 and 3.92, *p* < 0.005 and 0.001, respectively) and the left and right PPA (*t(34)* = 5.46 and 4.55, *p* < 0.001) in the scene *free-viewing* condition. The similarity was not significant in the scene *fixed-viewing* condition (*t(34)* = 0.13 ~ 1.63, *p* = 0.90 ~ 0.11 for the left and right HPC and PPA). Moreover, the similarity between the subsequent memory and fixation modulation effects was significantly stronger in the *free-* versus *fixed-viewing* condition (*t(34)* = 2.54 and 2.36, *p* = 0.008 and 0.012 for the left and right HPC; *t(34)* = 3.08 and 1.95, *p* = 0.002 and 0.03 for the left and right PPA, one-tailed; Figure 6*C*). Thus, in the *free-viewing* condition, the trial-wise number of fixations and subsequent memory performance shared variability at both the behavioral and the brain (individual voxel) level. When visual exploration was constrained in the *fixed-viewing* condition, the association between gaze fixations and memory at both levels was disrupted. For a complementary analysis that examined the interaction between the gaze fixation modulation effect and the subsequent memory effect on neural activity, please see the Supplementary Analysis and Figure S2 in the Supplementary Material.

We further examined whether the number of fixations predicted brain activity in the HPC and PPA even after controlling for effects of subsequent memory. We simultaneously entered the 2 modulator regressors (i.e., trial-wise number of fixations and subsequent memory) to the same parametric modulation analysis and examined the modulation effects of each regressor when controlling for the other regressor. Our parametric modulation ROI analysis showed that a greater number of gaze fixations indeed predicted stronger activation in the left and right PPA (*t(34)* = 3.96 and 5.35, *p* < 0.001. Figure 6*D*). The effect was not significant for the left and right HPC (*t(34)* = 0.29 and 0.73, *p* = 0.77 and 0.47, respectively) when the whole HPC ROIs were used. However, as shown in Figure 4*B*, the modulation effect of the number of fixations did not spread to the whole HPC. We therefore did a similar small volume correction analysis using the HPC template masks to correct for multiple comparisons, and thresholded the whole brain voxel-wise results with *p* = 0.005, 10-voxel extension (without corrections). The result revealed a cluster of voxels in both the left and right HPC that survived family-wise error correction at *p*_fwe-corr_ = 0.05 (for the left HPC: *p*_fwe-corr_ = 0.023, *p* < 0.0001, voxel size = 105 voxels with the peak voxel location = [−20, −32, −2]; for the right HPC: *p*_fwe-corr_ = 0.012, *p* < 0.0001, voxel size = 140 voxels with the peak voxel location = [28, −34, 4]). Brain section and surface images in Figure 6*E* and *F* illustrate the gaze fixation modulation effects in the PPA and HPC, as well as other brain regions, that occur above and beyond (and cannot be merely attributed to) subsequent memory effects. Moreover, the prediction of the numbers of gaze fixations in the *free-viewing* condition was stronger for scenes than the color-tile pictures for both the left and right PPA (*t(32)* = 6.80 and 5.95, *p* < 0.001) and the left and right HPC (*t(32)* = 2.83 and 3.44, *p* = 0.004 and < 0.001).

For completeness, we examined the subsequent memory effects after controlling for the modulation effect of the number of gaze fixations. Numerous brain regions, including the ventral and posterior medial temporal lobe regions, were modulated similarly by visual exploration and subsequent memory performance for the scenes under *free-viewing* conditions, even after the shared variance between the 2 variables was excluded (Figure 6*G*). To quantify the similarity between the 2 modulation effects at the whole-brain level, we calculated the modulation pattern similarity measure across all voxels in the brain between the fixation and subsequent memory effect using the same method as in Figure 6*C*. The similarity measure in the scene *free-viewing* condition was significantly larger than zero (*t(34)* = 2.93, *p* = 0.006; Figure 6*H*), and significantly greater than the similarity measure in the *fixed-viewing* condition (*t(34)* = 2.33, *p* = 0.013). Similarly, we calculated the whole-brain cross-voxel similarity between the 2 modulation effects when the shared variance between the 2 behavioral measures was retained. The similarity measure was again stronger in the *free-* than *fixed-viewing* condition (*t(34)* = 3.70, *p* < 0.001. The similarity measures were also greater when the shared variance was retained than when excluded (*p* < 0.001; Figure 6*H*), confirming that the shared variance at the behavioral level between the number of gaze fixations and subsequent memory was also reflected at the whole-brain cross-voxel level.

## Discussion

There is an intimate link between oculomotor behavior and memory (Ryan, Shen, and Liu 2020). The current study shows that robust relationships exist between naturalistic visual exploration and memory at both the behavioral and brain levels. Such findings lend strong support to the notion that gaze fixations provide the requisite units of information on which HPC binding processes operate to create new memories. This work provides the first non-invasive, comprehensive, and direct evidence of an oculomotor-memory system dependency in humans.

Visual exploration is positively related to recognition memory (Loftus 1972; Chan et al. 2011), and the restriction of viewing leads to a reduction in subsequent memory performance (Henderson et al. 2005; Damiano and Walther 2019). Findings from neurophysiology and neuroimaging have shown that oculomotor behavior modulates neural responses in regions that are critical for memory (Hoffman et al. 2013; Killian et al. 2015). When considered together, we predicted causal relationships between visual exploration, neural activity, and memory, such that restricting visual exploration would decrease neural responses in the HPC, broader medial temporal lobe, and along the ventral visual stream; change the functional connectivity between the HPC, PPA, and other cortical regions; and reduce subsequent memory. Here, we replicated our prior work using face stimuli (Liu et al. 2017, 2018) by showing that neural activity in the HPC, as well as in the PPA, were positively associated with the number of gaze fixations during free viewing of scenes. Critically, experimentally reducing visual exploration led to a reduction in neural activity all along the ventral visual stream, and up into the PPA and HPC, and reduced subsequent recognition memory. Gaze fixation modulation effects were not significant in the HPC or PPA for scenes under *fixed-viewing*, or for either of the scrambled color-tile picture conditions for which little information is available for extraction.

Given its well-established role in the processing of scenes (Aguirre et al. 1996; Epstein et al. 1999), we interrogated the functional connectivity of the PPA. When compared to *fixed-viewing*, *free-viewing* was associated with increased functional connectivity with the HPC, as well as with lateral and medial occipital cortex, and increased negative functional connectivity with regions such as the inferior parietal lobule, precuneus, anterior cingulate, lateral PFC, angular gyrus, and supramarginal gyrus. Reducing visual exploration therefore led to decreases in the relative strength of the functional connections across the network, and may have reduced information exchange between the oculomotor and medial temporal lobe networks (Shen et al. 2016; Ryan, Shen, Kacollja, et al. 2020a), resulting in declines in subsequent recognition memory. Thus, naturalistic viewing may coordinate responses (through correlated or potentially anti-correlated activity) across a broad network that includes regions responsible for the cognitive control of eye movements, perceptual processing of visual information, updating of spatial information, and memory (Sobotka et al. 2002; Purpura et al. 2003; Ryan, Shen, Kacollja, et al. 2020a).

The neural pattern of gaze modulation effects was similar to that of subsequent memory effects. The conjunction analysis confirmed that there were clusters in the PPA and the HPC where activity was predicted by the number of gaze fixations as well as subsequent memory. The cross-voxel brain activation patterns in the HPC, PPA, and even the whole brain that were associated with the number of gaze fixations were more similar to those associated with subsequent memory when scenes were encoded under *free-viewing*, compared to when scenes were encoded under *fixed-viewing* conditions. Importantly, clusters of neural activity in the HPC and PPA that were modulated by gaze fixations under *free-viewing* were evident even after controlling for the effect of subsequent memory. Together, these findings suggest that gaze fixations are related to the perceptual processing and binding functions of ventral visual stream, and HPC, respectively, above and beyond whether the resultant memory representations are available for conscious introspection. This is consistent with the purported role of the HPC in relational binding, in which elements are bound across space and time into a lasting, flexible representation, and may guide ongoing behavior in the moment, even in the absence of conscious awareness for the information contained therein (Cohen and Eichenbaum 1993; Eichenbaum and Cohen 2001; Olsen et al. 2012; Yonelinas 2013; Hannula et al. 2017). The added effect of gaze fixations on neural activity, beyond the effects of subsequent explicit recognition, is also consistent with work in which viewers who, in the absence of visual input, follow distinct scanpaths of other participants that had been elicited by viewing either faces or houses, show differential activation the fusiform face area and PPA, respectively (Wang et al. 2019). It is likely that lifelong experience viewing faces and houses creates stored exemplars of scanpaths that represent stimulus-specific viewing. Although future work is needed to explore how neural activity relates to the reproduction of specific scanpaths, the present findings suggest that, at the very least, gaze fixations contribute information that supports the development of memory. The sequence and location of those gaze fixations comprise the scanpath that may be part-and-parcel of any resultant memory trace (Noton and Stark 1971), even in the absence of conscious recognition for the viewers’ own scanpath (Clarke et al. 2017) or for the previously studied stimulus.

To be clear, this is not to say that visual exploration is completely independent of conscious recollection. Indeed, previous work has shown that the cumulative number of fixations is related to subsequent recognition (Loftus 1972; Olsen et al. 2016). Here, as noted in the Supplementary Material (Supplementary Analysis and Figure S2), there was a significant interaction of the gaze fixation and the subsequent memory modulation effects on activity in the right HPC. Results for the *free-viewing* condition for scenes indicated that the number of gaze fixations predicted activity in the right HPC better for well-recognized scenes compared to scenes that were not recognized as readily. Visual exploration therefore supports subsequent conscious recognition, perhaps through the increased engagement of the HPC. As noted above, however, the finding that visual exploration predicts HPC activity, even after controlling for effects of subsequent memory, suggests that the purpose of visual exploration, and of HPC activity more generally, go beyond its association to subsequent conscious appreciation.

Restricting visual exploration had a general effect in reducing associated neural activity, as neural responses were greater along the ventral visual stream and into the medial temporal lobe under *free-viewing* conditions for scenes and for color-tile images alike. This may suggest that eye movements simply have a faciliatory effect on neural activity, such that gaze fixations create a feedforward sweep of neural responses that prime the brain to efficiently process upcoming visual input. However, differences in neural activity for the *free-* versus *fixed-viewing* contrast were larger for scenes than for the color-tile images. Moreover, the cross-voxel modulation pattern of subsequent memory resembled that of visual exploration, which was stronger when scenes were viewed under the *free-* versus *fixed-viewing* condition. These findings suggest that visual exploration is related to the processing and encoding of informational content (Yarbus 1967; Henderson and Hayes 2017). An alternative account is that the reduction in neural responses during *fixed-* versus *free-viewing* reflects a decrease in saccadic suppression that would otherwise occur with movements of the eye (Ross et al. 2001). However, research points to an associated *reduction* of neural responses in visual processing regions such as V4 that occurs with eye movements (Kleiser et al. 2004). Here, the opposite occurred: there was an increase in neural activity along the visual processing stream associated with *free-viewing*, the condition for which greater saccade suppression should occur due to increased numbers of gaze fixations relative to *fixed-viewing*.

It is also unlikely that reductions in neural responses during *fixed-viewing* were effects of neural adaptation due to perceptual fading. Even during periods of steady fixation, there are small movements of the eyes (i.e., microssacades, ocular drift, tremors) that occur (Steinman et al. 1973; Martinez-Conde et al. 2004). In particular, microsaccades may be critical for improving visual acuity (Intoy and Rucci 2020), for allocating the fovea onto a target with precision (Putnam et al. 2005), for improving spatial resolution (Donner and Hemilä 2007; Rucci et al. 2007), and are associated with neural activity in the striate and extrastriate visual cortex (Leopold and Logothetis 1998; Snodderly et al. 2001). Moreover, even in cases of ocular paralysis, perceptual fading is not prominent (Whitham et al. 2011). It remains possible that some kind of adaptation occurred to a larger extent in the *fixed-viewing* condition because participants processed a more constrained region of the visual stimuli. Our results nonetheless show that in the *free-viewing* condition, the trial-wise number of fixations predicted PPA and HPC activity more strongly for scenes than scrambled color-tile images, and the viewing condition difference in this prediction strength (*free* > *fixed*) was also larger for scenes than scrambled color-tile images. Such findings suggest that the extent of visual exploration, the variable visual input gathered via multiple gaze fixations, and the information regarding direction and positions that are provided by the eye movement record itself, are important factors that each likely contribute to the present behavioral and neural findings. These results suggest that greater visual exploration enhances neural activity, and that restricting visual exploration impedes neural activity. Note that we do not consider this impediment to be synonymous with ‘active suppression’.

Whether the information garnered via eye movements makes a relatively larger or smaller contribution to the differential patterns of behavior and neural activity observed here than the eye movements themselves remains to be determined. At the very least, the match in eye movement task demands between the encoding and recognition phases may not be the critical determinant in memory performance observed in the present study. Here, for each trial in the retrieval task, the fixation cue, and thus the first fixation, were placed at the same location as in the encoding task. This should have helped viewers reinstate, at least to some extent, the encoding state. Participants could conceivably then enact the eye movement pattern that was optimal for themselves on each trial during retrieval, for example, by making more or fewer fixations that are directed to the same locations as in the encoding phase (*gaze reinstatement)* (Wynn et al. 2019). Prior research more definitively speaks to this issue. Damiano and Walther (Damiano and Walther 2019) demonstrated that hit rates for scenes were higher under *free-viewing* compared to *fixed-viewing* conditions during encoding, regardless of whether viewers engaged in *free-* or *fixed*-*viewing* at retrieval. At retrieval, *fixed viewing* was associated with a higher false alarm rate than *free viewing*, regardless of the viewing condition at encoding. These prior findings point to an important role for visual exploration during encoding for subsequent memory (Loftus 1972; Henderson et al. 2005; Damiano and Walther 2019) that is not merely due to effects of state-dependent learning. Here, we build on this prior work by providing causal evidence that the oculomotor and memory systems are intrinsically related at both the behavioral and neural level.

Another consideration for interpreting the present findings is whether *fixed-viewing* resulted in an increase in working memory demands in an effort to remain continually fixated that, in turn, altered the level of neural engagement and negatively affected memory. Prior work has shown that maintaining central fixation does not seem to increase working memory demands, as there was no reduction in n-back performance under *fixed-* versus *free-viewing* conditions (Armson et al. 2019). By contrast, performing a finger tapping task did interfere with n-back performance relative to *free-viewing*, as evidenced by a decrease in accuracy and an increase in response times (Armson et al. 2019). Thus, restricting visual exploration here likely resulted in a decrease in memory performance due to an inability to bind extracted visual information into a lasting memory representation, rather than due to an increased demand on working memory capacity.

We urge methodological caution for neuroimaging (fMRI, electroencephalography, magnetoencephalography) studies that require central fixation in an effort to reduce motion artifacts in the data; the findings here demonstrate how restriction of viewing behavior changes the amount of engagement within neural regions and across functional networks, and changes subsequent cognitive performance. This is not to say that central fixation would influence every study in such a manner. Viewing manipulations may have little to no effect when tasks employ simple stimuli and/or use simple task demands. However, maintaining central fixation may adversely affect the pattern of data that is observed when more complex stimuli are used, as seen here, or when the participant engages in more complex tasks (for further discussion, see (Ryan, Shen, and Liu 2020b)).

The present findings also call for future research that examines the level or type of information that is carried via gaze fixations on which the HPC (and broader medial temporal lobe) operates. Multiple theoretical accounts of HPC function purport that the HPC receives already-parsed information regarding objects, spatial locations, and temporal ordering for the purpose of then binding those elements into a coherent, lasting memory representation (Eichenbaum and Cohen 2001; Davachi 2006; Yonelinas 2013). However, oculomotor research typically considers the allocation of gaze fixations for the purpose of extracting information regarding lower-level features, such as luminance and contrast (Itti and Koch 2000). Evidence, especially the findings reported here, suggests that the HPC may bind visual information online, across gaze fixations (Hannula 2010; Liu et al. 2017; Voss et al. 2017). How, then, do we bridge this theoretical divide and understand the unit of information that gaze fixations carry to which the HPC is sensitive? Through its movements over space and time, gaze fixations inherently provide information regarding the spatial and temporal arrangements of distinct elements, and a plethora of research shows that cells in the HPC respond preferentially to particular places or temporal orders (Moser et al. 2008; MacDonald et al. 2013; Eichenbaum 2017). Alternatively, gaze fixations may serve to organize the sequential activity of neuronal assemblies in the HPC, from which properties of space and time may be derived (Buzsáki and Tingley 2018). While work remains to specifically address these issues, the present research provides key causal evidence supporting the notion that the oculomotor system is a unique effector system for memory (Ryan and Shen 2020) such that gaze fixations provide the fundamental units of information on which the binding functions of the HPC operate.

## Supplementary Material

Liu_Rosenbaum_Ryan_SceneViewing_SupplementaryMaterial_CCC_Accepted_tgaa054
