# Supplementary material for: Restricting Visual Exploration Directly Impedes Neural Activity, Functional Connectivity, and Memory
Source: Cereb Cortex Commun. 2020 Aug 25;1(1):tgaa054. doi: 10.1093/texcom/tgaa054 (PMC7595095; doi:10.1093/texcom/tgaa054)
Supplement: Liu_Rosenbaum_Ryan_SceneViewing_SupplementaryMaterial_CCC_Accepted_tgaa054 [file liu_rosenbaum_ryan_sceneviewing_supplementarymaterial_ccc_accepted_tgaa054.docx]

**Supplementary Material**

**Restricting visual exploration directly impedes neural activity, functional connectivity, and memory.**

Zhong-Xu Liu^1-3^, R. Shayna Rosenbaum^2-4^, Jennifer D. Ryan^2,3,5^

*^1^Department of Behavioral Sciences, University of Michigan-Dearborn, Dearborn, Michigan*

*^2^Rotman Research Institute, Baycrest, Toronto, ON, Canada*

*_3_Centre for Vision Research and Vision: Science to Applications (VISTA) Program, York University, Toronto, ON, Canada*

*^4^Department of Psychology, York University, Toronto, ON, Canada*

*^5^Departments of Psychology, Psychiatry, University of Toronto, Toronto, ON, Canada*

Corresponding Authors:

Zhong-Xu Liu

[zhongxu@umich.edu](mailto:zhongxu@umich.edu)

Table of Contents

[**Supplementary Figure S1:** ROI masks used in the present study 3](#_Toc45466758)

[**Supplementary Analysis and Figure S2:** *Fixation* x *subsequent memory* interaction effects on HPC and PPA activation during free viewing of scenes. 4](#_Toc45466759)

[**Table S1.** Mean memory performance and standard deviation (*SD*) in the *free-viewing* and *fixed-viewing* conditions. 5](#_Toc45466760)

[**Table S2.** Brain regions that showed stronger and weaker activation during the free-viewing, compared to fixed-viewing, of scenes. 6](#_Toc45466761)

[**Table S3.** Brain regions that showed stronger and weaker activation during the free-viewing, compared to fixed-viewing, of color-tile pictures. 7](#_Toc45466762)

[**Table S4.** Brain regions that showed stronger and weaker (free-viewing > fixed-viewing) viewing effect for the scenes compared to the color-tile pictures. 8](#_Toc45466763)

[**Table S5.** Brain regions for which activities were positively and negatively predicted by the trial-wise number of fixations for scenes in the free-viewing condition. 10](#_Toc45466764)

[**Table S6.** Regions showed stronger connectivity with the parahippocampal place area (PPA) when scenes were viewed in the free-, compared to the fixed-viewing condition. 12](#_Toc45466765)

[**Table S7.** Brain regions that showed subsequent memory effect for scenes under free-viewing condition. 14](#_Toc45466766)

### **Supplementary Figure S1:** ROI masks used in the present study


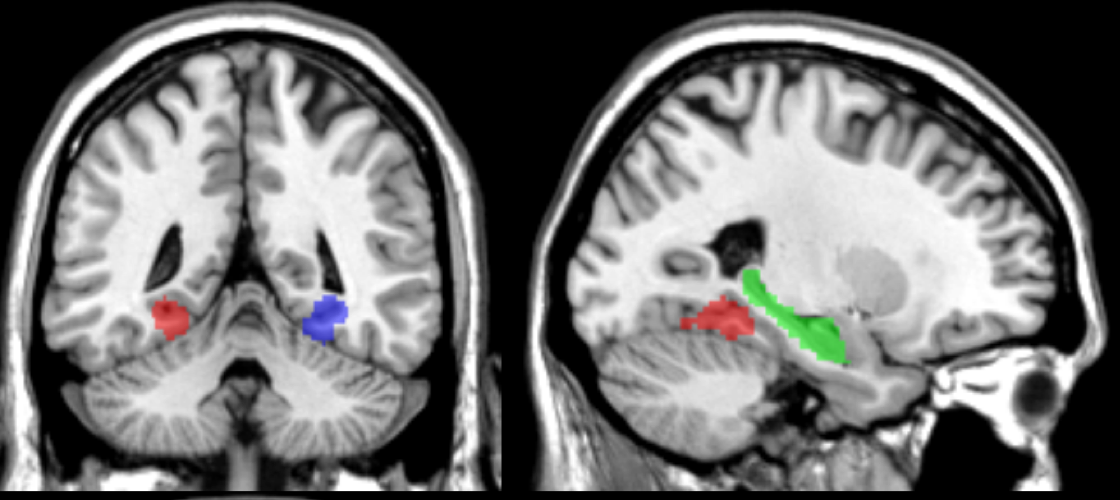


Green = hippocampus; red = left PPA; blue = right PPA. PPA = parahippocampal place area.

### **Supplementary Analysis and Figure S2:** *Fixation* x *subsequent memory* interaction effects on HPC and PPA activation during free viewing of scenes.

At the suggestion of an anonymous reviewer, we conducted another parametric modulation analysis with 3 modulators: the trial-wise number of fixations, subsequent recognition memory of each trial, and the interaction term between the two variables. For the interaction term, the product of the two variables were made as the third (i.e., last) modulator, after each variable was z-score transformed. The parametric modulation analysis procedure was identical to those in other parametric analyses reported in this study, except that 3 parametric modulators (instead of one or two) were entered. In this analysis, we only focused on the interaction effect in the free-viewing scene condition. Because we did not have an *a priori* hypothesis, a two-tailed t-test with alpha value of .05 was used for the ROI analysis.

We found that the right HPC showed a significant interaction effect, *t*(34) = 2.10, *p* = . 043. The left PPA approached significance, *t*(34) = 1.82, *p* = . 078. The interaction effect in the left HPC and right PPA was not significant, *t*(34) = 1.20/1.63, *p* = .24/.11, respectively. Brain images illustrating the effect in the HPC are presented below in Supplementary Figure S2 (only for illustration purposes). These results indicate that for better recognized scenes in the free-viewing condition, the number of fixations predicted brain activity in the right HPC, and to a lesser extent the left PPA, more strongly compared to the prediction for less-well recognized scenes. Although we did not make an *a priori* hypothesis about this effect, the result is consistent with other findings reported in this study and supports the idea that visual exploration may facilitate memory processing through its engagement of the medial temporal lobe, including the hippocampus.

(For completeness, we report that in the fixed-viewing scene condition, the left PPA showed a significant interaction effect, *t*(34) = 2.50, *p* = . 017. The right PPA and the left and right HPC did not show a significant effect, *t*(34) = 1.61, 1.15, and 1.53, *p* = .12, .26, and .13, respectively.)

**Supplementary Figure S2:** *Fixation* x *subsequent memory* interaction effects on HPC activation during free viewing of scenes (p < .05, no corrections; only for illustration purposes).


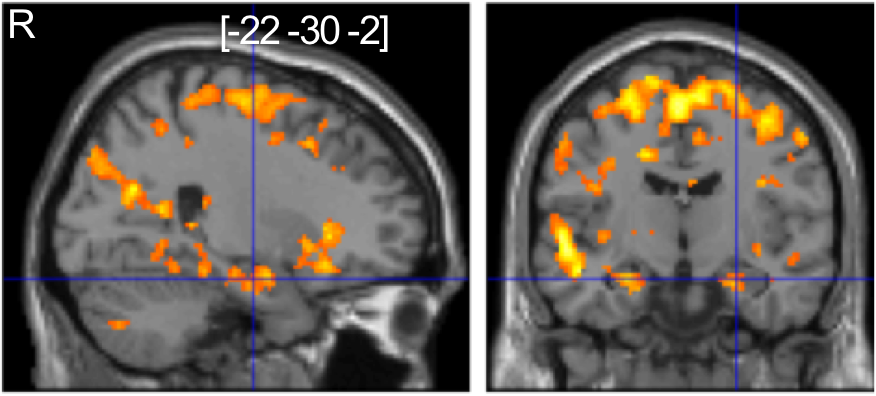


### **Table S1.** Mean memory performance and standard deviation (*SD*) in the *free-viewing* and *fixed-viewing* conditions.

|  | Scenes | | | | | Color-tile | | | |
| --- | --- | --- | --- | --- | --- | --- | --- | --- | --- |
|  | recognition memory | correctedaccuracy | | hit rate | false alarm rate | recognition memory | corrected accuracy | hit rate | false alarm rate |
| Free viewing | .46  (.32) | | 11.47%  (8.25%) | 46.71%  (14.99%) | 35.18%  (14.00%) | .22  (.30) | 0.74%  (7.41%) | 34.85%  (19.91%) | 34.10%  (20.10%) |
| Fixed viewing | .31  (.33) | 5.06%  (5.03%) | | 40.43%  (15.25%) | 35.18%  (14.00%) | .22  (.32) | 0.75%  (8.09%) | 34.55%  (19.35%) | 34.10%  (20.10%) |

***Notes*:**

a. Recognition memory was calculated by assigning 2 points to stimuli that were correctly recognized with high confidence, 1 point for those correctly recognized with low confidence, 0 points for previously viewed images endorsed as “new” with low confidence, and -1 point for previously viewed images endorsed as “new” with high confidence. A 2 x 2 repeated ANOVA (*free-/fixed-viewing* by scenes/color-tile pictures) showed that recognition memory was better for scenes than for color-tile pictures (*F*(1,35) = 6.92, *p* < .013), and higher for the images studied under *free-viewing* versus *fixed-viewing* instructions (*F*(1,35) = 27.23, *p* < .0001). This latter difference was significant only for the scenes (interaction effect: *F*(1,35) = 27.85, *p* < .0001);

b. Corrected accuracy = hit rate – false alarm rate. The false alarm rate is the same for the *free-* and *fixed-viewing* condition because the viewing condition is not relevant for the new pictures in the retrieval task. Similarly, a 2 x 2 repeated ANOVA (*free-/fixed-viewing* by scenes/color-tile pictures) showed that corrected accuracy was higher for scenes than for color-tile pictures (*F*(1,35) = 27.91, *p* < .0001), and higher for the images studied under *free-viewing* versus *fixed-viewing* instructions (*F*(1,35) = 21.35, *p* < .0001). This latter difference was significant only for the scenes (interaction effect: *F*(1,35) = 22.91, *p* < .0001);

c. We also examined whether the trial-wise number of fixations predicted recognition memory. Specifically, we ran a linear regression analysis for each condition in each run using the trial-wise number of fixations to predict recognition memory. The regression coefficient was averaged across runs for each participant. These coefficients from all participants were then subjected to independent t-tests to determine whether the mean was significantly higher than zero. This approach is identical to the two-level fMRI analysis approach used in this study.

d. We note that memory accuracy may have been relatively low in this experiment. This is due to the following factors: 1) a 1-hour delay between the encoding and retrieval phases, 2) old and new scenes were selected from the same sematic categories, and 3) large numbers of stimuli were used.

### **Table S2.** Brain regions that showed stronger and weaker activation during the free-viewing, compared to fixed-viewing, of scenes.

| **Anatomical areas** | **Cluster size** | ***t* value** | ***p* value** | **MNI coordinates** | | |
| --- | --- | --- | --- | --- | --- | --- |
|  |  |  |  | **x** | **y** | **z** |
| **Free-viewing > fixed-viewing** |  |  |  |  |  |  |
| Lingual_L_R* | 45470 | 12.15189 | 2.07E-14 | 22 | -44 | -8 |
| Frontal_Inf_Orb_R | 295 | 6.565433 | 7E-08 | 32 | 32 | -16 |
| Frontal_Inf_Tri_R | 352 | 4.642333 | 2.35E-05 | 56 | 24 | 28 |
| Insula_L | 186 | 4.416027 | 4.61E-05 | -32 | -22 | 20 |
| Cingulum_Ant_R | 76 | 4.322811 | 6.08E-05 | 6 | 2 | 26 |
| Postcentral_L | 80 | 3.582199 | 0.000513 | -62 | -10 | 34 |
| Postcentral_R | 48 | 3.560667 | 0.000544 | 62 | -6 | 36 |
| Frontal_Sup_L | 24 | 3.549678 | 0.000561 | -20 | 36 | 52 |
| Cerebelum_Crus2_R | 30 | 3.517169 | 0.000614 | 8 | -78 | -36 |
| Postcentral_R | 46 | 3.298407 | 0.001119 | 48 | -6 | 30 |
| Insula_R | 19 | 3.262465 | 0.001233 | 34 | -20 | 16 |
| Cingulum_Mid_R | 19 | 3.172249 | 0.00157 | 8 | -10 | 42 |
| Frontal_Sup_L | 14 | 3.165734 | 0.001598 | -16 | 64 | 18 |
| Temporal_Pole_Sup_L | 14 | 3.145887 | 0.001685 | -46 | 16 | -16 |
|  |  |  |  |  |  |  |
| **Fixed-viewing > free-viewing** |  |  |  |  |  |  |
| Parietal_Inf_R | 1685 | 9.128221 | 4.36E-11 | 56 | -52 | 46 |
| Parietal_Inf_L | 729 | 7.037532 | 1.71E-08 | -54 | -56 | 42 |
| Frontal_Inf_Oper_R | 373 | 6.334046 | 1.41E-07 | 50 | 4 | 18 |
| Cingulum_Post_R | 584 | 5.485443 | 1.84E-06 | 14 | -36 | 18 |
| Pallidum_R | 34 | 4.874105 | 1.17E-05 | 16 | 6 | -4 |
| Frontal_Mid_R | 478 | 4.441552 | 4.28E-05 | 40 | 28 | 38 |
| Insula_R | 61 | 4.395148 | 4.91E-05 | 30 | 16 | 12 |
| Temporal_Sup_L | 31 | 4.240839 | 7.73E-05 | -46 | -40 | 16 |
| Olfactory_R | 24 | 3.887782 | 0.000216 | 8 | 28 | -4 |
| Temporal_Mid_L | 31 | 3.7709 | 0.000301 | -56 | -68 | 10 |
| Frontal_Sup_R | 28 | 3.583863 | 0.00051 | 20 | 16 | 60 |
| Temporal_Mid_R | 24 | 3.512822 | 0.000622 | 54 | -26 | -16 |
| Pallidum_L | 10 | 3.465203 | 0.000709 | -12 | 6 | -2 |
| Temporal_Mid_L | 14 | 3.437713 | 0.000765 | -60 | -30 | -8 |
| SupraMarginal_R | 34 | 3.285998 | 0.001157 | 60 | -24 | 30 |
| Precentral_L | 10 | 3.034345 | 0.00226 | -46 | 0 | 20 |

*Note:* All clusters survived the threshold of *p* < .005, with 10 voxel extension, no correction. The names of the anatomical regions in the table, obtained using the AAL toolbox for SPM8, follow the automated anatomical labeling (AAL) template naming convention (Tzourio-Mazoyer et al., 2002). R/L - right/left hemisphere; Ant- anterior; Mid - middle; Sup - superior; Inf - inferior; Orb - orbital; Tri - triangularis. * indicates the voxel cluster that include voxels from the hippocampus.

### **Table S3.** Brain regions that showed stronger and weaker activation during the free-viewing, compared to fixed-viewing, of color-tile pictures.

| **Anatomical areas** | **Cluster size** | ***t* value** | ***p* value** | **MNI coordinates** | | |
| --- | --- | --- | --- | --- | --- | --- |
|  |  |  |  | **x** | **y** | **z** |
| **Free-viewing > fixed-viewing** |  |  |  |  |  |  |
| Cuneus_L_R* | 43928 | 10.1982 | 2.53E-12 | 14 | -84 | 20 |
| Frontal_Inf_Oper_R | 2675 | 5.89342 | 5.33E-07 | 46 | 16 | 30 |
| Amygdala_R | 207 | 5.54787 | 1.52E-06 | 34 | -2 | -24 |
| Frontal_Sup_Orb_L | 649 | 5.411448 | 2.31E-06 | -22 | 32 | -14 |
| ParaHippocampal_R | 17 | 4.954296 | 9.21E-06 | 16 | -14 | -24 |
| Temporal_Mid_L | 70 | 4.841744 | 1.29E-05 | -44 | -24 | -12 |
| Cerebelum_9_L | 82 | 4.661586 | 2.22E-05 | -14 | -46 | -48 |
| Frontal_Sup_Medial_R | 92 | 4.526411 | 3.32E-05 | 10 | 62 | 28 |
| Caudate_R | 86 | 4.39434 | 4.92E-05 | 4 | 20 | 4 |
| Frontal_Mid_L | 245 | 4.387761 | 5.02E-05 | -32 | 48 | 32 |
| Insula_R | 128 | 4.250593 | 7.52E-05 | 34 | -18 | 16 |
| Angular_L | 39 | 4.019954 | 0.000147 | -40 | -50 | 24 |
| Frontal_Sup_R | 160 | 4.00789 | 0.000153 | 16 | 40 | 46 |
| Postcentral_R | 120 | 3.79326 | 0.000283 | 66 | -8 | 26 |
| Insula_R | 38 | 3.764184 | 0.000307 | 34 | -4 | 12 |
| Putamen_R | 90 | 3.747869 | 0.000321 | 28 | 16 | 2 |
| Precentral_L | 53 | 3.721141 | 0.000347 | -52 | -4 | 24 |
| Temporal_Pole_Sup_R | 18 | 3.719921 | 0.000348 | 44 | 12 | -24 |
| Caudate_L | 31 | 3.501725 | 0.000641 | -14 | 30 | 2 |
| Thalamus_R | 23 | 3.483542 | 0.000674 | 22 | -10 | 2 |
| Postcentral_L | 51 | 3.426214 | 0.00079 | -48 | -20 | 24 |
| Frontal_Sup_Medial_R | 18 | 3.339045 | 0.001002 | 10 | 54 | 40 |
| Temporal_Sup_R | 40 | 3.321997 | 0.00105 | 46 | -30 | -2 |
| Frontal_Sup_L | 23 | 3.274441 | 0.001194 | -12 | 42 | 50 |
| Temporal_Mid_R | 23 | 3.225212 | 0.001363 | 60 | -42 | 4 |
| Putamen_L | 16 | 3.203533 | 0.001445 | -20 | 4 | 10 |
| Temporal_Sup_R | 20 | 3.187095 | 0.00151 | 64 | -42 | 20 |
| Insula_R | 23 | 3.172441 | 0.00157 | 28 | 18 | -18 |
| Frontal_Mid_L | 17 | 3.159482 | 0.001625 | -26 | 26 | 50 |
| Temporal_Sup_R | 24 | 3.11911 | 0.001808 | 44 | -18 | -8 |
| Temporal_Sup_L | 19 | 3.113383 | 0.001836 | -50 | -28 | 14 |
| **Fixed-viewing > free-viewing** |  |  |  |  |  |  |
| Precuneus_R | 27 | 3.3649 | 0.000934 | 22 | -42 | 16 |

*Note:* All clusters survived the threshold of p < .005, with 10 voxel extension, no correction. The names of the anatomical regions in the table, obtained using the AAL toolbox for SPM8, follow the automated anatomical labeling (AAL) template naming convention (Tzourio-Mazoyer et al., 2002). R/L - right/left hemisphere; Ant- anterior; Mid - middle; Sup - superior; Inf - inferior; Orb - orbital; Tri - triangularis. * indicates the voxel cluster that include voxels from the hippocampus.

### **Table S4.** Brain regions that showed stronger and weaker (free-viewing > fixed-viewing) viewing effect for the scenes compared to the color-tile pictures.

| **Anatomical areas** | **Cluster size** | ***t* value** | ***p* value** | **MNI coordinates** | | |
| --- | --- | --- | --- | --- | --- | --- |
|  |  |  |  | **x** | **y** | **z** |
| **Scenes > Color-tile** |  |  |  |  |  |  |
| Lingual_R* | 3818 | 9.124077 | 4.41E-11 | 26 | -44 | -8 |
| Lingual_L* | 3334 | 7.963201 | 1.14E-09 | -26 | -48 | -6 |
| Calcarine_L | 202 | 4.317955 | 6.16E-05 | -14 | -50 | 8 |
| Precuneus_R | 242 | 4.250809 | 7.51E-05 | 14 | -52 | 14 |
|  |  |  |  |  |  |  |
| **Scenes < Color-tile** |  |  |  |  |  |  |
| Cerebelum_Crus2_L | 178 | 5.365866 | 2.65E-06 | -18 | -74 | -40 |
| Parietal_Inf_L | 693 | 5.09892 | 5.95E-06 | -50 | -48 | 46 |
| Parietal_Sup_R | 1906 | 4.99295 | 8.19E-06 | 48 | -42 | 58 |
| Rolandic_Oper_R | 793 | 4.877378 | 1.16E-05 | 50 | 2 | 18 |
| Supp_Motor_Area_R | 571 | 4.632949 | 2.42E-05 | 16 | 18 | 60 |
| Temporal_Mid_L | 61 | 4.517921 | 3.41E-05 | -60 | -32 | -10 |
| Frontal_Mid_R | 767 | 4.420299 | 4.55E-05 | 44 | 48 | 4 |
| Heschl_R | 513 | 4.367033 | 5.33E-05 | 36 | -26 | 6 |
| Supp_Motor_Area_R | 59 | 4.347381 | 5.65E-05 | 14 | -20 | 56 |
| Supp_Motor_Area_L | 49 | 4.22608 | 8.08E-05 | -12 | 2 | 66 |
| Frontal_Mid_Orb_L | 368 | 4.225111 | 8.1E-05 | -36 | 54 | -4 |
| Cingulum_Mid_R | 232 | 4.01296 | 0.00015 | 20 | -10 | 48 |
| Precentral_L | 129 | 3.99754 | 0.000157 | -26 | -16 | 54 |
| Frontal_Mid_R | 435 | 3.899613 | 0.000208 | 42 | 28 | 40 |
| Cingulum_Mid_L | 51 | 3.88171 | 0.000219 | -14 | -8 | 38 |
| Cerebelum_7b_L | 66 | 3.789446 | 0.000286 | -38 | -52 | -44 |
| Frontal_Sup_L | 103 | 3.786231 | 0.000288 | -12 | 42 | 34 |
| Supp_Motor_Area_R | 27 | 3.668746 | 0.000402 | 6 | -6 | 62 |
| Angular_L | 84 | 3.634419 | 0.000443 | -58 | -58 | 26 |
| Insula_L | 10 | 3.606453 | 0.000479 | -34 | 12 | 14 |
| Temporal_Mid_L | 13 | 3.606255 | 0.000479 | -52 | -44 | -12 |
| Cerebelum_6_L | 24 | 3.558782 | 0.000547 | -18 | -50 | -30 |
| Temporal_Sup_L | 11 | 3.555089 | 0.000553 | -46 | -40 | 14 |
| Frontal_Mid_R | 111 | 3.461617 | 0.000716 | 36 | 40 | 34 |
| Frontal_Mid_L | 55 | 3.439514 | 0.000761 | -24 | 6 | 40 |
| Temporal_Pole_Sup_R | 22 | 3.410617 | 0.000824 | 44 | 10 | -24 |
| Frontal_Sup_L | 55 | 3.379754 | 0.000897 | -14 | 14 | 54 |
| Precentral_L | 61 | 3.357984 | 0.000952 | -48 | 6 | 20 |
| Insula_L | 29 | 3.348258 | 0.000977 | -26 | 18 | 6 |
| Frontal_Inf_Oper_R | 11 | 3.31719 | 0.001064 | 24 | 16 | 30 |
| Frontal_Mid_L | 41 | 3.305507 | 0.001098 | -44 | 30 | 32 |
| Cuneus_R | 34 | 3.303799 | 0.001103 | 8 | -76 | 24 |
| Precentral_L | 11 | 3.301655 | 0.001109 | -44 | -4 | 38 |
| Frontal_Mid_L | 22 | 3.231787 | 0.001339 | -34 | 20 | 36 |
| Frontal_Sup_R | 14 | 3.17175 | 0.001573 | 20 | 36 | 30 |
| Cuneus_R | 12 | 3.154787 | 0.001645 | 10 | -78 | 36 |
| Cuneus_L | 10 | 3.149628 | 0.001668 | -12 | -76 | 20 |
| Frontal_Mid_L | 13 | 3.080441 | 0.002003 | -38 | 6 | 36 |
| Temporal_Pole_Sup_L | 14 | 3.053262 | 0.002151 | -48 | 8 | -2 |
| Postcentral_R | 19 | 3.004731 | 0.002441 | 62 | -18 | 30 |
| Cingulum_Mid_R | 10 | 2.996816 | 0.002492 | 8 | 36 | 32 |
| Lingual_L | 11 | 2.966526 | 0.002695 | -12 | -60 | -4 |
| Precentral_R | 13 | 2.932666 | 0.002941 | 34 | -24 | 56 |

*Note:* All clusters survived the threshold of p < .005, with 10 voxel extension, no correction. The names of the anatomical regions in the table, obtained using the AAL toolbox for SPM8, follow the automated anatomical labeling (AAL) template naming convention (Tzourio-Mazoyer et al., 2002). R/L - right/left hemisphere; Ant- anterior; Mid - middle; Sup - superior; Inf - inferior; Orb - orbital; Tri - triangularis. * indicates the voxel cluster that include voxels from the hippocampus.

### **Table S5.** Brain regions for which activities were positively and negatively predicted by the trial-wise number of fixations for scenes in the free-viewing condition.

| **Anatomical areas** | **Cluster size** | ***t* value** | ***p* value** | **MNI coordinates** | | |
| --- | --- | --- | --- | --- | --- | --- |
|  |  |  |  | **x** | **y** | **z** |
| **Positive prediction** |  |  |  |  |  |  |
| Calcarine_L | 18321 | 11.01328 | 4.66E-13 | 2 | -88 | -2 |
| Hippocampus_L* | 292 | 5.418666 | 2.45E-06 | -22 | -30 | -2 |
| Parietal_Sup_L | 393 | 4.921602 | 1.09E-05 | -24 | -66 | 52 |
| Caudate_L | 411 | 4.821737 | 1.46E-05 | -22 | -8 | 22 |
| Cerebelum_9_L | 60 | 4.619454 | 2.66E-05 | -16 | -42 | -46 |
| Thalamus_R | 34 | 4.427182 | 4.69E-05 | 22 | -14 | -2 |
| Cerebelum_9_R | 68 | 4.241445 | 8.06E-05 | 16 | -50 | -46 |
| Cerebelum_8_L | 72 | 3.853914 | 0.000246 | -24 | -64 | -44 |
| Amygdala_L | 10 | 3.486204 | 0.000686 | -20 | -4 | -20 |
| Vermis_10 | 60 | 3.409834 | 0.000845 | -4 | -52 | -30 |
| Hippocampus_R* | 33 | 3.391569 | 0.000888 | 38 | -12 | -24 |
| Frontal_Inf_Orb_L | 19 | 3.363371 | 0.000959 | -30 | 32 | -18 |
| Occipital_Mid_L | 13 | 3.198458 | 0.001492 | -22 | -56 | 32 |
| Putamen_L | 11 | 3.159043 | 0.001656 | -22 | 6 | 6 |
| Lingual_R | 12 | 3.055618 | 0.002172 | 10 | -50 | -2 |
|  |  |  |  |  |  |  |
| **Negative prediction** |  |  |  |  |  |  |
| Angular_R | 2510 | 6.022786 | 4.02E-07 | 40 | -68 | 48 |
| Precuneus_L | 1638 | 5.752032 | 9.04E-07 | -4 | -56 | 42 |
| Occipital_Mid_L | 689 | 5.709919 | 1.03E-06 | -40 | -76 | 40 |
| Frontal_Sup_R | 4854 | 5.448484 | 2.24E-06 | 18 | 30 | 42 |
| Insula_R | 973 | 5.333827 | 3.17E-06 | 30 | 22 | -6 |
| Temporal_Mid_R | 879 | 5.240514 | 4.19E-06 | 62 | -30 | -12 |
| Frontal_Mid_L | 483 | 5.027965 | 7.9E-06 | -28 | 18 | 54 |
| Insula_L | 409 | 4.779616 | 1.66E-05 | -32 | 18 | -10 |
| Cingulum_Mid_L | 246 | 4.420831 | 4.77E-05 | -8 | 4 | 36 |
| SupraMarginal_L | 384 | 4.272937 | 7.36E-05 | -54 | -24 | 14 |
| Temporal_Mid_L | 57 | 4.119872 | 0.000115 | -66 | -48 | -2 |
| Pallidum_R | 22 | 3.997447 | 0.000163 | 18 | 2 | 0 |
| Temporal_Mid_L | 238 | 3.955889 | 0.000184 | -64 | -24 | -10 |
| Cingulum_Ant_R | 46 | 3.933193 | 0.000196 | 6 | 18 | 20 |
| Frontal_Mid_L | 335 | 3.910862 | 0.000209 | -38 | 52 | 2 |
| Cerebelum_6_L | 10 | 3.854185 | 0.000245 | -24 | -50 | -30 |
| Cingulum_Mid_L | 203 | 3.806512 | 0.000281 | -4 | -22 | 38 |
| Parietal_Inf_L | 56 | 3.721111 | 0.000357 | -50 | -32 | 46 |
| Precentral_R | 20 | 3.604576 | 0.000495 | 24 | -14 | 52 |
| Cerebelum_6_L | 12 | 3.582896 | 0.000525 | -14 | -62 | -26 |
| Frontal_Inf_Tri_L | 83 | 3.565554 | 0.000551 | -42 | 30 | 24 |
| Vermis_4_5 | 22 | 3.429425 | 0.000801 | 4 | -52 | -14 |
| Precentral_L | 11 | 3.408819 | 0.000847 | -46 | 2 | 20 |
| Postcentral_L | 60 | 3.407134 | 0.000851 | -20 | -30 | 64 |
| Cingulum_Ant_R | 18 | 3.336626 | 0.001031 | 6 | 30 | 8 |
| Postcentral_R | 27 | 3.266626 | 0.001244 | 32 | -40 | 70 |
| Precentral_L | 23 | 3.208074 | 0.001454 | -20 | -18 | 74 |
| Cerebelum_4_5_R | 14 | 3.16168 | 0.001645 | 22 | -48 | -26 |
| Temporal_Sup_L | 16 | 3.095545 | 0.001957 | -44 | 0 | -8 |
| Postcentral_R | 11 | 3.000727 | 0.002505 | 24 | -44 | 60 |
| Parietal_Sup_L | 13 | 2.917289 | 0.003103 | -24 | -46 | 72 |

*Note:* All clusters survived the threshold of p < .005, with 10 voxel extension, no correction. The names of the anatomical regions in the table, obtained using the AAL toolbox for SPM8, follow the automated anatomical labeling (AAL) template naming convention (Tzourio-Mazoyer et al., 2002). R/L - right/left hemisphere; Ant- anterior; Mid - middle; Sup - superior; Inf - inferior; Orb - orbital; Tri - triangularis. * indicates the voxel cluster that include voxels from the hippocampus.

### **Table S6.** Regions showed stronger connectivity with the parahippocampal place area (PPA) when scenes were viewed in the free-, compared to the fixed-viewing condition.

| **Anatomical areas** | **Cluster size** | ***t* value** | ***p* value** | **MNI coordinates** | | |
| --- | --- | --- | --- | --- | --- | --- |
|  |  |  |  | **x** | **y** | **z** |
| **Left PPA** |  |  |  |  |  |  |
| Calcarine_R | 11599 | 9.499591 | 1.6E-11 | 8 | -82 | 0 |
| Parietal_Sup_R | 521 | 5.834378 | 6.38E-07 | 26 | -60 | 50 |
| Supp_Motor_Area_L | 285 | 5.720036 | 9.03E-07 | -2 | 8 | 72 |
| Hippocampus_R* | 137 | 5.714706 | 9.18E-07 | 26 | -24 | -4 |
| Hippocampus_L* | 67 | 5.322309 | 3.02E-06 | -20 | -20 | -10 |
| Caudate_R | 175 | 5.31743 | 3.07E-06 | 14 | 28 | -2 |
| Frontal_Inf_Oper_R | 96 | 4.766433 | 1.62E-05 | 40 | 12 | 26 |
| Frontal_Inf_Orb_L | 90 | 4.481842 | 3.79E-05 | -46 | 34 | -12 |
| Frontal_Mid_Orb_R | 17 | 4.396707 | 4.88E-05 | 38 | 38 | -16 |
| Parietal_Sup_L | 199 | 4.286442 | 6.76E-05 | -22 | -64 | 58 |
| Frontal_Sup_Medial_L | 65 | 4.145779 | 0.000102 | -4 | 66 | 26 |
| Precentral_L | 117 | 4.132133 | 0.000106 | -46 | 0 | 58 |
| Cerebelum_10_L | 92 | 4.114556 | 0.000112 | -24 | -38 | -40 |
| Caudate_L | 49 | 4.113555 | 0.000112 | -20 | 12 | 24 |
| Cerebelum_6_L | 27 | 3.794541 | 0.000282 | -30 | -48 | -26 |
| Cerebelum_8_R | 25 | 3.762975 | 0.000308 | 26 | -38 | -48 |
| SupraMarginal_L | 45 | 3.736125 | 0.000332 | -46 | -38 | 30 |
| Putamen_L | 28 | 3.693142 | 0.000375 | -26 | -24 | 4 |
| Occipital_Mid_L | 138 | 3.65078 | 0.000423 | -22 | -92 | 0 |
| Temporal_Inf_L | 13 | 3.604872 | 0.000481 | -44 | -32 | -12 |
| Occipital_Sup_L | 15 | 3.593966 | 0.000496 | -24 | -78 | 42 |
| Frontal_Inf_Tri_R | 53 | 3.585249 | 0.000508 | 48 | 26 | 18 |
| Vermis_3 | 36 | 3.575103 | 0.000523 | 4 | -32 | -4 |
| Temporal_Inf_R | 24 | 3.561748 | 0.000543 | 44 | -12 | -26 |
| Fusiform_R | 10 | 3.461729 | 0.000716 | 20 | 14 | -44 |
| Frontal_Inf_Orb_L | 14 | 3.450275 | 0.000739 | -34 | 36 | -10 |
| Caudate_R | 38 | 3.320849 | 0.001053 | 12 | -6 | 24 |
| Thalamus_L | 14 | 3.317949 | 0.001061 | -2 | -14 | 16 |
| Frontal_Inf_Tri_R | 12 | 3.291466 | 0.00114 | 56 | 38 | 16 |
| Caudate_L | 20 | 3.232363 | 0.001337 | -12 | 22 | 12 |
| Precentral_R | 10 | 3.214593 | 0.001402 | 56 | 12 | 40 |
| Precuneus_R | 10 | 3.19248 | 0.001488 | 10 | -66 | 68 |
| Cerebelum_9_R | 10 | 3.134873 | 0.001734 | 14 | -44 | -44 |
|  |  |  |  |  |  |  |
| **Right PPA** |  |  |  |  |  |  |
| Occipital_Inf_R | 14985 | 11.201 | 2.01E-13 | 40 | -80 | -4 |
| Hippocampus_L* | 124 | 6.395288 | 1.17E-07 | -22 | -24 | -6 |
| Frontal_Mid_Orb_L | 73 | 6.105368 | 2.81E-07 | -34 | 36 | -14 |
| Thalamus_R* | 130 | 5.618295 | 1.23E-06 | 24 | -22 | -4 |
| Supp_Motor_Area_R | 303 | 5.472495 | 1.92E-06 | 4 | 12 | 66 |
| Frontal_Inf_Tri_R | 156 | 5.258107 | 3.67E-06 | 54 | 32 | 0 |
| Caudate_R | 157 | 5.157291 | 4.98E-06 | 24 | -30 | 24 |
| Cerebelum_7b_L | 41 | 4.814644 | 1.4E-05 | -6 | -76 | -40 |
| Precentral_L | 101 | 4.73292 | 1.79E-05 | -48 | -4 | 50 |
| Cingulum_Ant_R | 263 | 4.66837 | 2.18E-05 | 18 | 34 | 6 |
| Precentral_R | 25 | 4.481076 | 3.8E-05 | 38 | -14 | 38 |
| Lingual_R | 60 | 4.404961 | 4.77E-05 | 6 | -30 | -4 |
| Caudate_L | 66 | 4.392404 | 4.95E-05 | -18 | 32 | 0 |
| Frontal_Inf_Tri_L | 215 | 4.298525 | 6.53E-05 | -54 | 34 | 2 |
| Frontal_Inf_Tri_R | 152 | 4.132302 | 0.000106 | 38 | 18 | 20 |
| Frontal_Inf_Orb_R | 51 | 4.024198 | 0.000146 | 34 | 34 | -22 |
| Cerebelum_10_L | 28 | 3.851404 | 0.000239 | -18 | -40 | -44 |
| Temporal_Pole_Sup_R | 35 | 3.740122 | 0.000329 | 34 | 22 | -26 |
| Frontal_Sup_Medial_L | 36 | 3.688224 | 0.000381 | -2 | 66 | 18 |
| Precentral_L | 13 | 3.680204 | 0.000389 | -26 | -12 | 48 |
| Parietal_Sup_L | 131 | 3.643467 | 0.000432 | -20 | -52 | 52 |
| Rolandic_Oper_L | 14 | 3.632471 | 0.000445 | -48 | -16 | 22 |
| Temporal_Pole_Sup_L | 14 | 3.59922 | 0.000489 | -46 | 24 | -26 |
| Rolandic_Oper_R | 11 | 3.448575 | 0.000743 | 48 | -6 | 8 |
| Frontal_Sup_L | 18 | 3.365325 | 0.000933 | -28 | -2 | 68 |
| Occipital_Mid_L | 14 | 3.291732 | 0.001139 | -48 | -76 | 8 |
| Caudate_L | 10 | 3.247858 | 0.001283 | 0 | 12 | 4 |
| Precentral_R | 14 | 3.207404 | 0.00143 | 24 | -30 | 68 |
| Frontal_Mid_R | 12 | 3.189278 | 0.001501 | 46 | 0 | 58 |
| Precentral_R | 17 | 3.185396 | 0.001516 | 52 | 2 | 50 |
| Supp_Motor_Area_L | 11 | 3.178357 | 0.001545 | -10 | -6 | 64 |
| Frontal_Sup_Medial_L | 16 | 3.165672 | 0.001598 | -2 | 52 | 44 |
| Temporal_Inf_L | 13 | 3.150594 | 0.001664 | -34 | 4 | -36 |

*Note:* All clusters survived the threshold of p < .005, with 10 voxel extension, no correction. The names of the anatomical regions in the table, obtained using the AAL toolbox for SPM8, follow the automated anatomical labeling (AAL) template naming convention (Tzourio-Mazoyer et al., 2002). R/L - right/left hemisphere; Ant- anterior; Mid - middle; Sup - superior; Inf - inferior; Orb - orbital; Tri - triangularis. * indicates the voxel cluster that include voxels from the hippocampus.

### **Table S7.** Brain regions that showed subsequent memory effect for scenes under free-viewing condition.

| **Anatomical areas** | **Cluster size** | ***t* value** | ***p* value** | **MNI coordinates** | | |
| --- | --- | --- | --- | --- | --- | --- |
|  |  |  |  | **x** | **y** | **z** |
| **Remembered > forgotten** |  |  |  |  |  |  |
| Occipital_Sup_R* | 5385 | 7.44754 | 6.1E-09 | 26 | -68 | 38 |
| Precentral_R | 742 | 6.510548 | 9.43E-08 | 46 | 4 | 32 |
| Occipital_Mid_L* | 4352 | 6.189985 | 2.44E-07 | -34 | -88 | 22 |
| Cerebelum_9_L | 82 | 5.200029 | 4.72E-06 | -14 | -46 | -48 |
| Thalamus_R | 64 | 5.022265 | 8.04E-06 | 18 | -30 | 2 |
| Frontal_Inf_Oper_L | 262 | 4.41166 | 4.9E-05 | -44 | 8 | 28 |
| Caudate_R | 33 | 4.158551 | 0.000103 | 26 | 8 | 18 |
| Thalamus_R | 44 | 4.098952 | 0.000122 | 2 | -14 | 4 |
| Frontal_Inf_Orb_L | 26 | 3.866363 | 0.000237 | -32 | 36 | -16 |
| Precuneus_L | 40 | 3.813852 | 0.000275 | -16 | -50 | 10 |
| Insula_R | 25 | 3.652804 | 0.000432 | 46 | 6 | 6 |
| Insula_L | 35 | 3.652133 | 0.000433 | -34 | -2 | -14 |
| Putamen_L | 14 | 3.492128 | 0.000675 | -22 | -6 | 12 |
| Cerebelum_9_L | 53 | 3.384715 | 0.000905 | -2 | -52 | -34 |
| Frontal_Inf_Orb_R | 36 | 3.316984 | 0.001087 | 28 | 32 | -12 |
| Caudate_L | 34 | 3.251357 | 0.001296 | -8 | 8 | 18 |
| Supp_Motor_Area_R | 34 | 3.236993 | 0.001347 | 8 | 12 | 54 |
| Putamen_L | 12 | 3.159378 | 0.001655 | -20 | 6 | 6 |
| Thalamus_R | 23 | 3.049127 | 0.002209 | 10 | -20 | 6 |
|  |  |  |  |  |  |  |
| **Forgotten > remembered** |  |  |  |  |  |  |
|  |  |  |  |  |  |  |
| Cingulum_Post_R | 6388 | 7.447096 | 6.11E-09 | 2 | -48 | 30 |
| Angular_R | 3762 | 6.425117 | 1.21E-07 | 52 | -52 | 36 |
| Cingulum_Ant_R | 9569 | 6.187421 | 2.46E-07 | 4 | 44 | 26 |
| Insula_R | 253 | 6.055739 | 3.65E-07 | 32 | 20 | -10 |
| Angular_L | 4483 | 5.897652 | 5.85E-07 | -46 | -56 | 38 |
| Precentral_L | 152 | 4.863593 | 1.29E-05 | -26 | -14 | 66 |
| Temporal_Pole_Sup_R | 32 | 4.170016 | 9.92E-05 | 56 | 10 | -12 |
| Postcentral_R | 71 | 4.159064 | 0.000102 | 20 | -44 | 64 |
| Cerebelum_Crus2_L | 66 | 4.106555 | 0.000119 | -24 | -86 | -34 |
| Insula_L | 89 | 4.104821 | 0.00012 | -32 | 16 | -10 |
| Cingulum_Ant_L | 50 | 4.059104 | 0.000137 | -4 | 18 | 20 |
| Cingulum_Post_R | 80 | 3.978703 | 0.000172 | 10 | -36 | 12 |
| Lingual_L | 80 | 3.96981 | 0.000177 | -12 | -56 | -4 |
| Lingual_R | 119 | 3.684425 | 0.000396 | 12 | -58 | -6 |
| Supp_Motor_Area_R | 11 | 3.478273 | 0.000701 | 12 | -22 | 56 |
| Frontal_Inf_Tri_R | 48 | 3.472286 | 0.000712 | 48 | 20 | 2 |
| Precentral_L | 10 | 3.47227 | 0.000712 | -34 | -4 | 38 |
| Frontal_Mid_L | 37 | 3.402615 | 0.000862 | -38 | 38 | 22 |
| Temporal_Mid_R | 11 | 3.331515 | 0.001045 | 52 | -4 | -28 |
| Postcentral_L | 35 | 3.263992 | 0.001253 | -48 | -24 | 56 |
| Temporal_Pole_Mid_R | 17 | 3.206362 | 0.001461 | 46 | 16 | -30 |

*Note:* All clusters survived the threshold of *p* < .005, with 10 voxel extension, no correction. The names of the anatomical regions in the table, obtained using the AAL toolbox for SPM8, follow the automated anatomical labeling (AAL) template naming convention (Tzourio-Mazoyer et al., 2002). R/L - right/left hemisphere; Ant- anterior; Mid - middle; Sup - superior; Inf - inferior; Orb - orbital; Tri - triangularis. * indicates the voxel cluster that include voxels from the hippocampus.
